# Therapeutic Perspective of Vitamin C and Its Derivatives

**DOI:** 10.3390/antiox8080247

**Published:** 2019-07-26

**Authors:** Andrijana Meščić Macan, Tatjana Gazivoda Kraljević, Silvana Raić-Malić

**Affiliations:** Department of Organic Chemistry, Faculty of Chemical Engineering and Technology, University of Zagreb, Marulićev trg 20, 10000 Zagreb, Croatia

**Keywords:** l-ascorbic acid, vitamin C, butenolide, antitumor, anticancer, antiviral, antioxidant

## Abstract

l-Ascorbic acid (ASA), vitamin C, is a ubiquitous carbohydrate-like compound that has an essential role in a number of cellular processes, such as collagen synthesis, cellular oxidation, and various hydroxylation reactions. ASA is a biomolecule of critical importance for protection of cellular components against oxidative damage caused by toxic free radicals and other reactive oxygen species (ROS) that are involved in the development of various types of chronic diseases. Vitamin C has a switchover role from being an antioxidant in physiological conditions to a prooxidant under pathologic conditions. Moreover, some l-ascorbic acid derivatives exhibit strong and selective antitumor and antiviral activity. This review emphasizes the advances on diverse and potent biological profiles of l-ascorbic acid and its derivatives, and their perspective in the development of new bioactive chemical entities in the future. The work is primarily addressed at antioxidant, anticancer, and antiviral potencies of l-ascorbic acid and compounds containing its butenolide structural motif.

## 1. Introduction: Vitamin C and Its Antioxidant and Antitumor Activity

**Figure 1:**
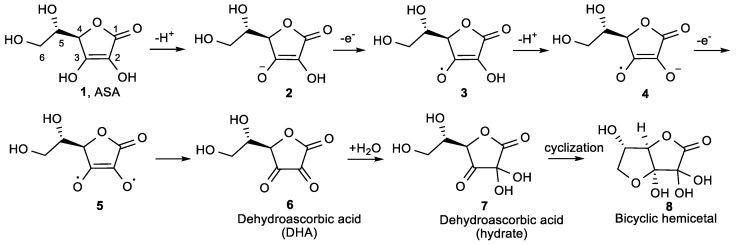
The mechanism for the ionization and oxidation of l-ascorbic acid (ASA) to the inactive dehydroascorbic acid **6** and bicyclic hemiacetal **8**.

Since the discovery of vitamin C (l-ascorbic acid, ASA, 1, Figure 1) acting as a powerful anti-scorbutic agent, the number of its biological properties is continually expanding [1]. It is known that ascorbate, a conjugate base of l-ascorbic acid, is an antioxidant and free radical scavenger, as well as a cofactor required for key enzyme reactions [2]. Vitamin C has been shown to suppress free radical generation and attenuate oxidative damage resulting from the reactive oxygen species (ROS) [2,3]. Since many chronic diseases such as cancer, diabetes, atherosclerosis, cardiovascular diseases, inflammation, neurodegenerative diseases, and aging are associated with oxidative stress [4,5], some studies of l-ascorbic acid have been focused on its ROS scavenging activity to prevent oxidative stress-related diseases [6,7]. It has also been found that ASA exhibits prooxidant activity. This behavior can be explained by the very good reducing ability of ascorbate [8], which can reduce catalytic metals such as Fe^3+^ to Fe^2+^ and Cu^2+^ to Cu^+^, increase the prooxidant chemistry of these metals, and facilitate the generation of ROS. The reduced metals can react with oxygen, reducing it to superoxide radical (which dismutates to H_2_O_2_ and O_2_), and can also react with hydrogen peroxide to form hydroxyl radicals via Fenton-type reaction [9,10,11,12,13]. Due to the action of antioxidant enzymes such as catalase and glutathione peroxidase, normal cells are not damaged by ROS. On the other hand, ROS can cause cell death, because many cancer cells have lower levels of antioxidant enzymes compared to normal cells.

It is worth mentioning that ascorbate is present in cytosol, chloroplast, vacuoles, mitochondria, and the extracellular matrix of plants [14]. Together with glutathione, reduced nicotinamide adenine dinucleotide phosphate (NADPH), and enzymes linking these metabolites, ascorbate participates in a cycle of reactions (Foyer-Halliwell-Asada pathway) which have an important role in removal of H_2_O_2_ and the protection of different cell compartments against oxidative damage. In the first step of the cycle, the enzyme ascorbate peroxidase, using ascorbate as the electron donor, reduces H_2_O_2_ to water. Ascorbate is later recovered in the cycle [15,16].

Vitamin C is an essential, water-soluble micronutrient that exists predominantly as the ascorbate anion under physiological pH conditions, arising from the completely dissociated 3-hydroxyl group. ASA contains a lactone ring with electron rich 2-en-2,3-diol-1-one moiety (Figure 1). As a reducing agent and electron donor antioxidant, ASA can undergo two consecutive one-electron oxidation reactions and deprotonation of both hydroxyl groups at positions 2 and 3, resulting in the formation of dehydroascorbic acid (DHA, **6**, Figure 1).

Hydroxyl group at position 3 in **1**, as a vinylogous carboxylic acid, ionizes first, and ASA is ionized at physiological pH to its highly polar, water-soluble 3-monoanion (**2**) [17]. At pH 7, 99.9% of vitamin C is present as the anion **2**, therefore the antioxidant chemistry of vitamin C is the chemistry of the anion **2** [18]. Loss of one electron from **2** gives an ascorbate radical **3**, which is resonance stabilized. Because of the low reduction potential of the ascorbate radical/ascorbate couple (E^0^ = 282 mV), almost every oxidazing radical formed in the biological system causes one electron oxidation of **2**, resulting in the ascorbate radical **3** [19]. The ionization of the 2-OH group of **3**, that is more facile than the ionization of the 2-OH group of the monoanion **2**, subsequently affords **4**. Another electron donation of **4** gives the diradical **5,** that then rearranges to non-toxic oxidized form, dehydroascorbic acid **6**. In this process, ASA donates two protons and two electrons to terminate the radical chain reactions. Dehydroascorbic acid **6** forms a hydrate **7** that cyclizes to the bicyclic hemiacetal **8**, which is structurally similar to glucose and, therefore, is transported to cells by glucose transporters (GLUTs) [20].

ASA is taken up by most cell types via a high affinity/low capacity mechanism through sodium-dependent vitamin C transporter (SVCT-1 and 2), or as dehydroascorbic acid (DHA) being accumulated via sodium-independent facilitative GLUTs, followed by intracellular reduction [18,21,22]. While the reduced form of ascorbate is dominant in the plasma of healthy humans, DHA is present at a very low level, indicating that ascorbate is taken up and accumulated in cells primarily by SVCTs.

Driven by the connection between oxidative stress and acute respiratory distress syndrome (ARDS) [23], as well as reports that subnormal ascorbate concentrations are common in patients with sepsis, and that ascorbate levels in plasma correlate with survival and inversingly with multiple organ failure [24,25], several case studies have been performed. Encouraging results of intravenous vitamin C administration, either alone or in combination with other antioxidants, as an adjunctive therapy to treat sepsis-induced ARDS have been reported [26,27,28,29,30].

In the early 1970s, the two-time Nobel Prize-winning chemist Linus Pauling reported that high doses of vitamin C reduced cancer by acting as an antioxidant [31]. L. Pauling and E. Cameron demonstrated that intravenous administration of vitamin C, followed by oral use, led to an increased rate of survival of cancer patients [32,33]. On the contrary, other clinical studies have shown that vitamin C has a low antitumor effect [34,35].

Cancer therapy using high-dose vitamin C has a controversial history, which inspired further studies that significantly contributed to the understanding of the contradictory clinical data, explained by differences in the route of administration [36]. 

In connection with the prooxidant role of ascorbate, which requires pharmacological (millimolar) rather than physiological (high micromolar) concentrations [8,37,38], a growing number of phase I/II clinical trials are reevaluating intravenous infusion of vitamin C to treat various cancers [39,40,41].

**Figure 2:**
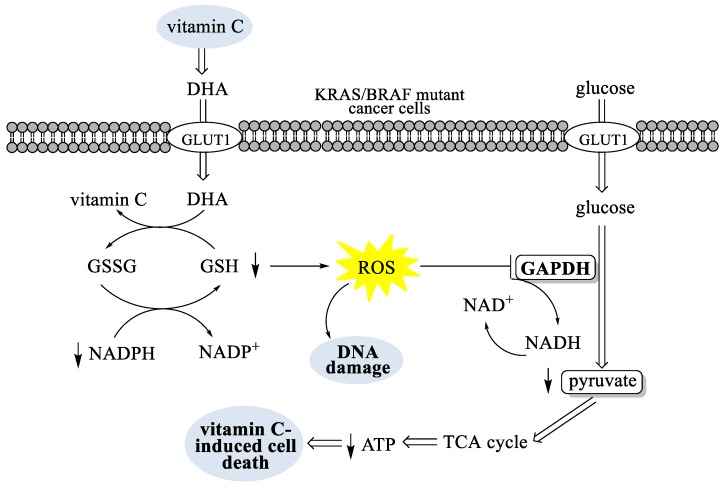
The mechanistic overview of vitamin C toxicity in cancer cells with KRAS and BRAF mutations. GSSG: oxidized form of glutathione; NADP^+^: nicotinamide adenine dinucleotide phosphate; NADH: reduced nicotinamide adenine dinucleotide; NAD+: oxidized nicotinamide adenine dinucleotide; TCA: citric acid cycle also known as the tricarboxylic acid or the Krebs cycle. DHA: dehydroascorbic acid; GLUT1: glucose transporter 1; KRAS: kirsten rat sarcoma viral oncogene; BRAF: v-raf murine sarcoma viral oncogene homolog B; GSH: reduced form of glutathione; GSSG: oxidized form of glutathione; NADPH: reduced nicotinamide adenine dinucleotide phosphate; NADP^+^: nicotinamide adenine dinucleotide phosphate; ROS: reactive oxygen species; GAPDH: glyceraldehyde 3-phosphate dehydrogenase; NADH: reduced nicotinamide adenine dinucleotide; NAD+: oxidized nicotinamide adenine dinucleotide; TCA: citric acid cycle also known as the tricarboxylic acid or the Krebs cycle; ATP: adenosine 5′-triphosphate.

According to recent reports, vitamin C selectively kills KRAS (kirsten rat sarcoma viral oncogene) - and BRAF (v-raf murine sarcoma viral oncogene homolog B)-driven colorectal cancer cells (CRCs) by inducing oxidative stress, suppressing glycolysis and the subsequent energy crisis, and cell death. As mentioned, cells take up the oxidized form of vitamin C, DHA, through glucose transporters such as GLUT1, where DHA is reduced to vitamin C by glutathione (GSH). The increased uptake of DHA into the KRAS or BRAF mutant cancer cells leads to the rapid conversion of DHA to vitamin C, resulting in the depletion of GSH and NADPH and an increase in ROS that cause DNA damage (Figure 2). Increases in ROS have been shown to activate the oxidative pentose phosphate pathway, increasing in glycolytic intermediates upstream of glyceraldehyde 3-phosphate dehydrogenase (GAPDH) and decreasing in metabolites downstream of the GAPDH reaction. Inhibition of GAPDH decreases the formation of glycolytic adenosine 5′-triphosphate (ATP) and pyruvate, leading to an energetic crisis that triggers cell death [42].

These results provide a mechanistic rationale for exploring the therapeutic use of vitamin C for CRCs with KRAS or BRAF mutations, but it is still unclear whether the results observed in evaluated cell culture and mouse studies can translate to human tumors [42]. It has also been found that vitamin C therapy can be a possible treatment for pancreatic cancer, another typically lethal cancer driven by KRAS [43]. The recent case report of the use of pharmacologic ascorbic acid (PASA) as a sole treatment in a patient with poorly differentiated stage IV pancreatic ductal adenocarcinoma (PDA) showed that PASA should be taken into consideration, along with previous research in cell, animal, and clinical experiments when designing future treatment trials [44]. Recent studies have demonstrated that an intravenous high-dose of PASA induces cytotoxicity and oxidative stress selectively in pancreatic cancer cells vs. normal cells, suggesting a promising new role of ascorbate as a therapeutic agent [45].

Ascorbate treatment also induced cell death, caused by the nuclear translocation of apoptosis-inducing factor (AIF) in human breast cancer cells [46]. A high-dose of vitamin C was shown to inhibit the growth of breast adenocarcinoma MCF7 and colon cancer HT29 cells by blocking the energy flux in glucose catabolism and the citric acid (TCA) cycle, which subsequently caused insufficient ATP formation and cell death [47]. Kim et al. recently reported that a high-dose of vitamin C selectively induces DNA damage of cancer stem cells, suggesting a potential application of vitamin C in the treatment of cancer stem cells [48].

Recent findings indicate that intracellular ascorbate decreases HIF-1α (hypoxia-inducible factor) protein levels, resulting in reduced HIF-1 activity [18,49,50,51,52,53,54]. As a result of intratumoral hypoxia, as well as some genetic alterations, HIF-1 and HIF-2 are overexpressed in human cancers, making them an important target for cancer therapy. Under normoxic conditions, the α-subunit of HIF is degraded by oxygen-dependent hydroxylation of its proline and asparagine residues with HIF-hydroxylase enzymes, resulting in proteasomal degradation of HIF-α. It is believed that ASA acts as a cofactor for iron-dependent HIF-hydroxylase enzymes, possibly by keeping the iron of the enzyme in the active Fe^2+^ state, and therefore, the absence of ASA leads to HIF activation [55,56].

**Figure 3:**
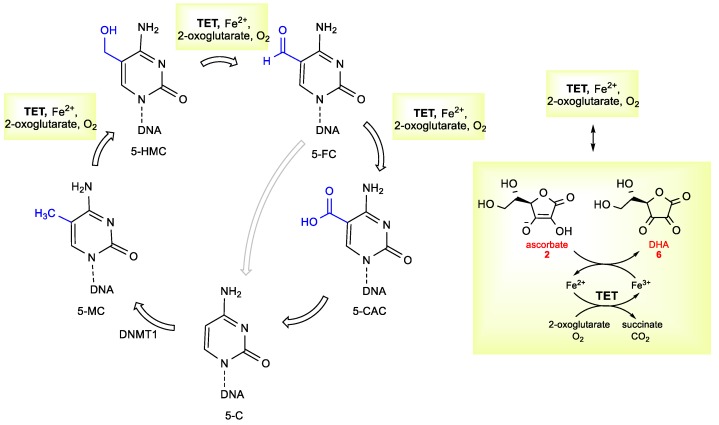
The mechanistic overview of ascorbate in DNA demethylation. 5-HMC: 5-hydroxymethylcytosine; 5-MC: 5-methylcytosine; DNMT1: DNA methyltransferase 1; 5-C: 5-cytosine; 5-CAC: 5-carboxylcytosine; 5-FC: 5-formylcytosine; TET: ten-eleven translocation dioxygenases.

Furthermore, recent progress in epigenetics identified a new role of ascorbate in the epigenetic modulation of gene activity [57,58,59]. DNA methylation is a key epigenetic mechanism that results in the heritable silencing of genes, which has led to an interest in inhibiting DNA methylation as a therapeutic strategy for treating cancer. It has been found that ascorbate serves as a cofactor for ten-eleven translocation (TET) dioxygenases, which catalyze the oxidation of 5-methylcytosine (5-MC) into 5-hydroxymethylcytosine (5-HMC), further to 5-formylcytosine (5-FC) and 5-carboxylcytosine (5-CAC) (Figure 3) [60]. 5-FC and 5-CAC are then transformed into 5-cytosine (5-C). The methylation of 5-C by DNA methyltransferase (DNMT1) to give 5-MC completes a cycle of DNA methylation-demethylation reactions. 

By regulating the epigenetic mechanism, ascorbate could be involved in embryonic development, postnatal development, aging, cancer, and other diseases. In light of this, it was found that ASA promoted the TET-mediated generation of 5-HMC in mouse and human cells [61,62], suggesting that vitamin C may contribute to epigenetic regulation of gene expression by functioning as a cofactor for TET enzymes, leading to DNA demethylation. Indeed, it was found that vitamin C, but not other antioxidants, acts as a direct regulator of TET activity and DNA methylation in mouse embryonic stem (ES) cells, thus indicating its potential application for treating cancers driven by aberrant DNA methylation in the clinic [63].

## 2. Antioxidant Properties of l-Ascorbic Acid Derivatives

Lipophilicity is an important determinant of antioxidant activity of ASA derivatives, as it regulates mobility and distribution through the membrane phospholipid bilayer. It was found that the susceptibility of vitamin C to thermal and oxidative degradation, together with its poor liposolubility, make it difficult to maintain its physiological value over a long period of time and to penetrate cell membrane [64]. Therefore, structural modifications of ASA by the introduction of the lipophilic moieties have led to diverse ASA derivatives with increased thermal and oxidative stability [64,65]. Some reports have described the effects of modified ASA derivatives on oxidative damage of biological molecules, especially nucleic acid and lipid membranes [66,67,68] by free radicals produced from the Fenton reaction [11,12]. Depending on concentrations, the effects of ASA derivatives on lipid peroxidation could be found to be pro- or antioxidant, while the prooxidant effects might be important in vivo depending on the availability of catalytic metal ions.

### 2.1. Lipophilic ASA Derivatives with Modified Hydroxyl Groups

**Figure 4:**
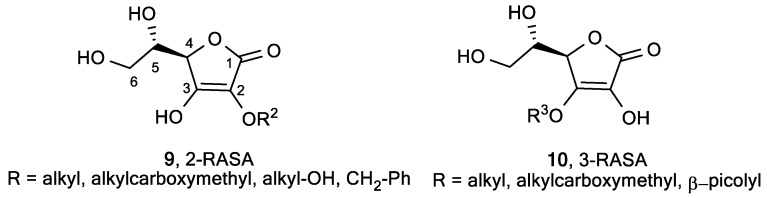
The lipophilic 2-*O*-alkylated (2-RASA) and 3-*O*-alkylated (3-RASA) ASA derivatives.

A series of 2-*O*-alkylated (2-RASA, **9**) [69] and 3-*O*-alkylated (3-RASA, **10**) [68] ASA derivatives, in which the hydroxyl group at positions 2 and 3 was substituted with alkyl groups of various lengths, were synthesized to act as radical scavengers for ROS and free radicals (Figure 4). Their redox potentials and inhibitory effects on lipid peroxidation in rat liver microsomes were evaluated (Table 1).

The redox potentials of the 3-RASA derivatives (**10**) were increased above the potential for ASA itself (Table 1). When observing the one electron reduction potential of free radicals (peak potential) for a molecule to be a thermodynamically good antioxidant, it is desirable to be highly reducing with a lower value of the peak potential (at the bottom of the pecking order for oxidizing radicals), which makes it more willing to donate an electron [70]. While 2-RASA derivatives (**9**) showed the same antioxidant activity as ASA, 3-RASA compounds exhibited lower antioxidant properties. Although 3-*O*-dodecylascorbic acid (**10a**) and 3-*O*-(decylcarboxymethyl) ascorbic acid (**10b**) differed in their redox potentials, they both markedly inhibited lipid peroxidation in rat liver microsomes. Structure-activity relationship (SAR) studies have demonstrated that the anti-lipid peroxidation activity of 2-RASA and 3-RASA compounds was strongly dependent upon their lipophilicity. It was observed that both a long alkyl chain and the electron donating activity of the enolic hydroxyl group in 2-RASA and 3-RASA are required in the suppression of lipid peroxidation. These results also indicate that ASA analogues with an appropriate lipophilicity can easily penetrate through phospholipid bilayers and act as free-radical quenchers that protect against the lipid peroxidation of the biomembrane. The experiments in vivo strongly suggest that the prevention of lipid peroxidation by ROS could diminish post-ischemic tissue injury in the rat ischemia-reperfusion model. 

Liu et al. synthesized ASA derivatives with hydroxyl groups at positions 2 and 3, modified by simple alkyl groups, and performed DNA damage experiments to determine which OH groups contribute the most to the oxidative damage (Figure S1) [66]. While some ascorbic acid derivatives selectively cleaved plasmid DNA, other derivatives slightly oxidatively damaged plasmid DNA. The derivatives that have been methylated at 2-OH or 3-OH can hardly exert oxidative damage on plasmid DNA, confirming that the free hydroxyl groups of ASA contribute to the oxidative damage of DNA.

**Figure 5:**
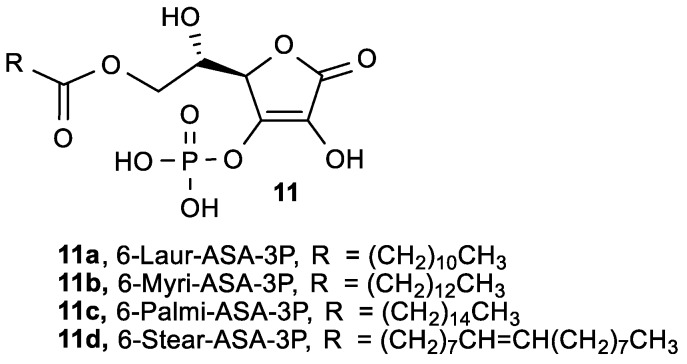
6-Acyl-ASA-3P derivatives.

A series of 6-*O*-acyl-l-ascorbic acid-3-*O*-phosphates (6-Acyl-ASA-3P, **11**, Figure 5) was synthesized to act as radical scavengers for ROS, and free radicals and their cytotoxicity on highly metastatic human lung carcinoma (95-D) cells were investigated (Table 2) [71]. 

All synthesized compounds showed stronger ROS scavenging ability and cytotoxicity in comparison to ASA. The optimal length of the side chain in position 6 is 12 carbon atoms in 6-Laur-ASA-3P (**11a**), which proved to be the best candidate for the development of a new lipophilic ASA derivative with antioxidant and antiproliferative effects. Furthermore, Liu et al. investigated the inhibition of tumor invasion in vivo for 6-Laur-ASA-3P (**11a**), 6-Myri-ASA-3P (**11b**), and 6-Stear-ASA-3P (**11d**) [72]. It was found that intraperitoneal administration of 6-Laur-ASA-3P (**11a**) (75 mg/kg) decreased the number of metastatic nodules by 62% and elevated the survival rate of C57BL/6 mice compared to the control group.

Similar to the 6-Acyl-ASA-3-phosphate derivatives, 2-phosphate analogue of ASA with a 6-palmitate chain (6-Palmi-ASA-2P, Figure S2) was found to be superior to ASA, and exhibited a protective role on the damage of PC12 cells induced by hydrogen peroxide [73]. The potency of the protective role of 6-Palmi-ASA-2P on PC12 cells correlated with its ROS scavenging activity. Fan et al. showed that 6-Palmi-ASA-2P (Figure S2) could effectively protect the human umbilical cord vein endothelial cells against hydrogen peroxide and *tert*-butyl hydroperoxide-induced cytotoxicity, and exhibited no cytotoxicity within the tested concentration range [74]. 

**Figure 6:**
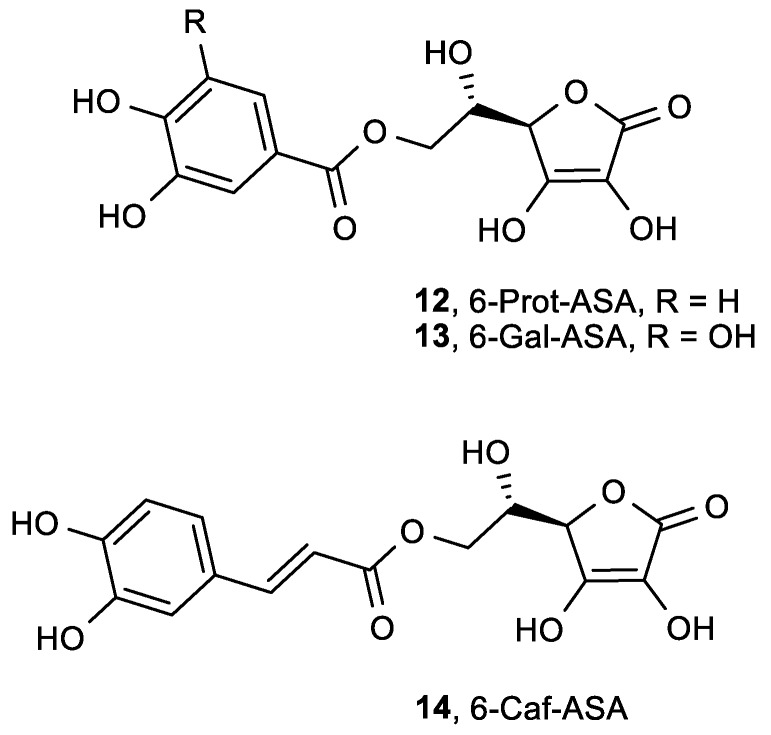
ASA phenolic esters.

In order to synthesize ASA derivatives that maintain the antioxidant properties of ASA with decreased side effects on platelet function compared to ASA, and keeping in mind the inhibitory effect of some phenolic compounds on platelet aggregation stimulated by thrombin, new ASA phenolic esters—l-ascorbyl-6-protocatechuate (6-Prot-ASA, **12**), l-ascorbyl-6-gallate (6-Gal-ASA, **13**) and l-ascorbyl-6-caffeate (6-Caf-ASA, **14**)—were synthesized by Lopez et al. (Figure 6) [75]. 

Compounds **12**–**14** showed greater radical scavenging activity when compared to ASA (Table 3). These compounds also altered platelet calcium homeostasis in response to thrombin. 6-Prot-ASA (**12**) was found to be the ascorbyl phenolic ester with the strongest antioxidant properties but weakest antiaggregant actions. Therefore, its use as an antioxidant, with the aim of preventing thrombotic alteration in patients that need antioxidant therapy, could be safer than the use of other derivatives.

Kato et al. synthesized a 5-*O*-modified 4-iodobenzylated ASA derivative (Figure S3) with a radical scavenging activity in the DPPH assay similar to that of ASA, indicating its potential use in imaging of ascorbate bioactivity in the brain [76].

### 2.2. Glucoside ASA Derivatives

**Figure 7:**
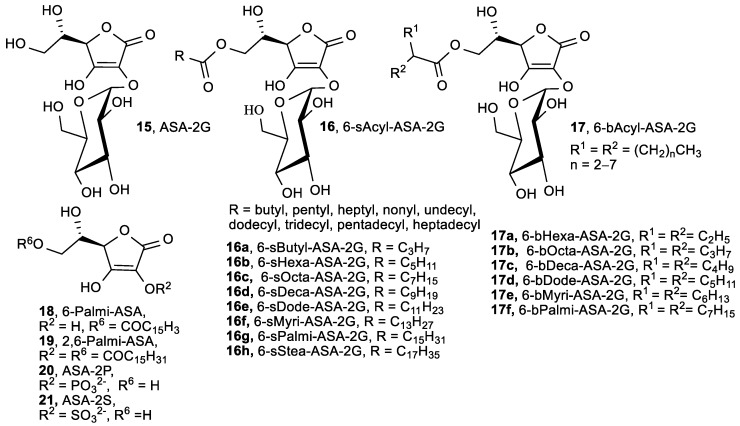
Glucoside ASA derivatives as potential radical scavengers.

From glucoside ASA derivatives, 2-*O*-*α*-d-glucopyranosyl-l-ascorbic acid (ASA-2G, **15**) and 6-*O*-acyl-2-*O*-*α*-d-glucopyranosyl-l-ascorbic acid (6-Acyl-ASA-2G, **16**, **17**) (Figure 7) exhibited radical scavenging activity, as well as anti-scorbutic activity and collagen synthesis regulation, in vivo after enzymatic hydrolysis by *α*-glucosidase and esterase [77]. Besides the aforementioned activity of ASA-2G (**15**) [78], its diverse biological effects have been expanded to enhanced B-cell differentiation [79], hepatocyte growth factor production [80], human keratinocytes protection from UV-B [81], collagen synthesis by dermal fibroblasts in vitro [82], and inhibition of melanin synthesis [83]. ASA-2G has also been proven to have a longer lasting protection of free radical-induced cytotoxicity when compared to ASA [84]. Additionally, Taniguchi et al. showed that pretreatment with ASA-2G (**15**) promoted the proliferation of normal human dermal fibroblasts (NHDF) and protected against cellular senescence and cell damage induced by hydrogen peroxide [85]. 

Xiao et al. showed that 6-Palmi-ASA (**18**) protects human lymphocytes, preferentially over ascorbate, against X-ray-induced DNA damage, lipid peroxidation, and protein carbonylation [86]. 6-Palmi-ASA (**18**), but not ASA, has been shown to act as a non-competitive inhibitor of lipoxygenase 5-LOX (IC_50_ = 2.5 µM) and soybean 15-LOX (IC_50_ = 10.3 µM), suggesting a crucial role of the long hydrophobic alkyl chain for the improved binding affinity of compounds to LOX [87]. LOX are the key enzymes involved in the biosynthesis of leukotrienes and ROS, which are involved in the pathophysiology of inflammatory disorders.

6-Palmi-ASA (**18**) inhibited 5-LOX more strongly than known LOX inhibitors phenidone and nordihydroguaiaretic acid. In contrast, 2,6-Palmi-ASA (**19**), ASA-2P (**20**), and ascorbic acid 2-sulfate (ASA-2S, **21**) exhibited negligible scavenging activity. A series of 6-Acyl-ASA-2G possessing a straight-acyl chain length from C_4_ to C_18_ (6-sAcyl-ASA-2G, **16a**–**h**), and compounds with branched-acyl chain length from C_6_ to C_16_ (6-bAcyl-ASA-2G, **17a**–**f**) [88], exhibited lower radical scavenging activity than that of ASA (Table 4). The conversion of a straight-acyl chain into a branched-acyl chain had no effect on radical scavenging ability. Furthermore, 6-Acyl-ASA-2G tended to increase the scavenging activity with increasing length of their acyl group [77,89]. 

**Figure 8:**
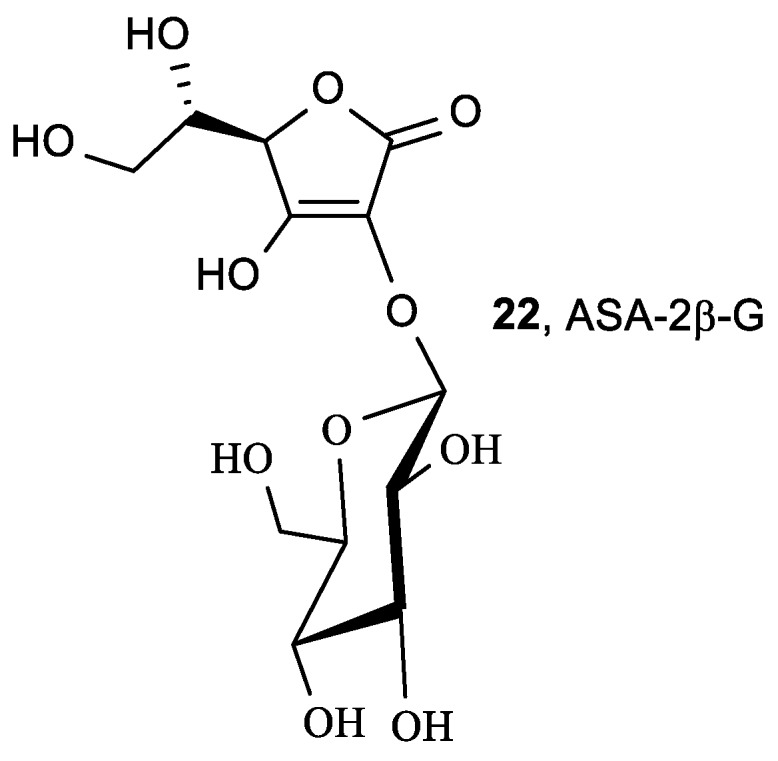
The pro-vitamin C derivative ASA-2*β*-G.

Takebayashi et al. studied the inhibitory effects of 2-*O*-substituted ASA derivatives—ASA-2G (**15**), ASA-2P (**20**), and ASA-2S (**21**) (Figure 7)—on AAPH-induced oxidative hemolysis of sheep erythrocytes, and compared the results with those of ASA and other antioxidants [90]. The 2-*O*-substituted ASA derivatives, without being enzymatically converted to ASA, showed inhibitory effects equivalent to, or stronger than, the inhibitory effects of ASA on AAPH-induced oxidation of sheep erythrocyte membrane proteins and hemolysis. While ASA-2G (**15**, Figure 7) contains a d-glucose moiety connected to the C-2 via α-glucoside linkage, 2-*O*-*β*-d-glucopyranosyl-l-ascorbic acid (ASA-2*β*-G, **22**, Figure 8) is a natural derivative of vitamin C isolated from Goji berry (*Lycium barbarum* L.) fruit that contains a d-glucose moiety at the C-2 via *β*-glucoside linkage [91]. 

Radical scavenging assays demonstrated that ASA-2*β*-G (**22**) was capable of scavenging DPPH and hydroxyl peroxide and inhibiting H_2_O_2_-induced hemolysis better than ASA [92]. ASA-2*β*-G and ASA had similar hydroxyl radical scavenging abilities. However, ASA-2*β*-G was unable to scavenge superoxide anion radicals, and scavenging of nitrite (NO_2_^−^) was lower than for ASA. Moreover, in vivo studies in mice demonstrated that ASA-2*β*-G (**22**) protected the liver against carbon tetrachloride-induced acute liver injury. Takebayashi et al. showed that ASA-2*β*-G slowly and continuously scavenged DPPH radicals and 2,2′-azinobis(3-ethylbenzothiazoline-6-sulfonic acid) radical cation (ABTS^+^) in the same reaction profiles as ASA-2G (**15**), whereas ASA quenched these radicals instantly [93]. 

### 2.3. Conjugates of Vitamin C and E

**Figure 9:**
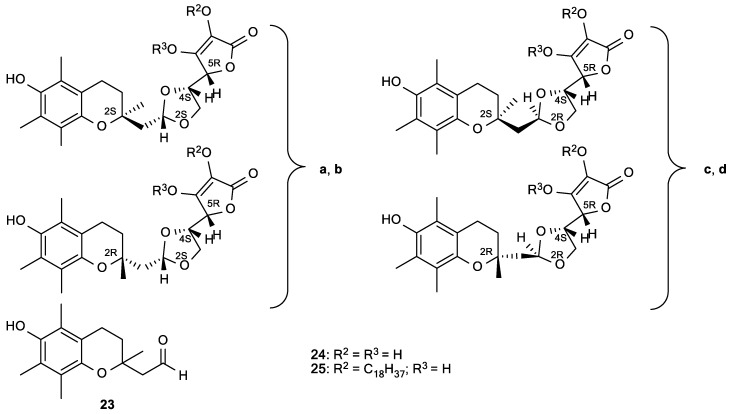
The antioxidant agents derived from a molecular combination of the l-ascorbic acid and α-tocopherol.

An important effect of ascorbate is its synergistic interaction with lipid-soluble vitamin E, which is known as a primary antioxidant in low-density lipoprotein (LDL) and lipid membrane oxidation. It was observed that vitamin C increased the antioxidant potency of vitamin E, suggesting that vitamin C regenerates vitamin E from the formed vitamin E radicals [18]. The phenolic hydroxyl group of tocopherol and the formed tocopheroxyl radical are at the membrane-water interface near the phospholipid polar head groups, enabling its recycling by water-soluble vitamin C [70]. Based on this, Manfredini et al. prepared a new type of synergistic antioxidant agents (**24** and **25**) by a combination of vitamin C and E (Figure 9) [94]. These compounds inhibited production of AAPH-induced malondialdehyde (MDA) in a peroxyl radical-dependent lipoperoxidation assay (Table 5). Compounds **24a**–**d** that present the hybrids of the *α*-tocopherol analogue **23** and ASA exhibited greater antioxidant efficacy than the natural antioxidants, with IC_50_ in the range of 7–12 µM. The configuration at the dioxolane and chromane ring, and the substitution at position 2 of the lactone moiety, influenced the antioxidant activities. The superior antioxidant activity may be caused by the concomitant presence of the lactone and *α*-tocopheryl residue, which can facilitate a radical trapping activity in both hydrophilic and lipophilic compartments of plasma and, thus, enhance the antioxidant potential by scavenging of ROS and lipoperoxyl radicals [94].

### 2.4. Butenolide Derivatives

**Figure 10:**
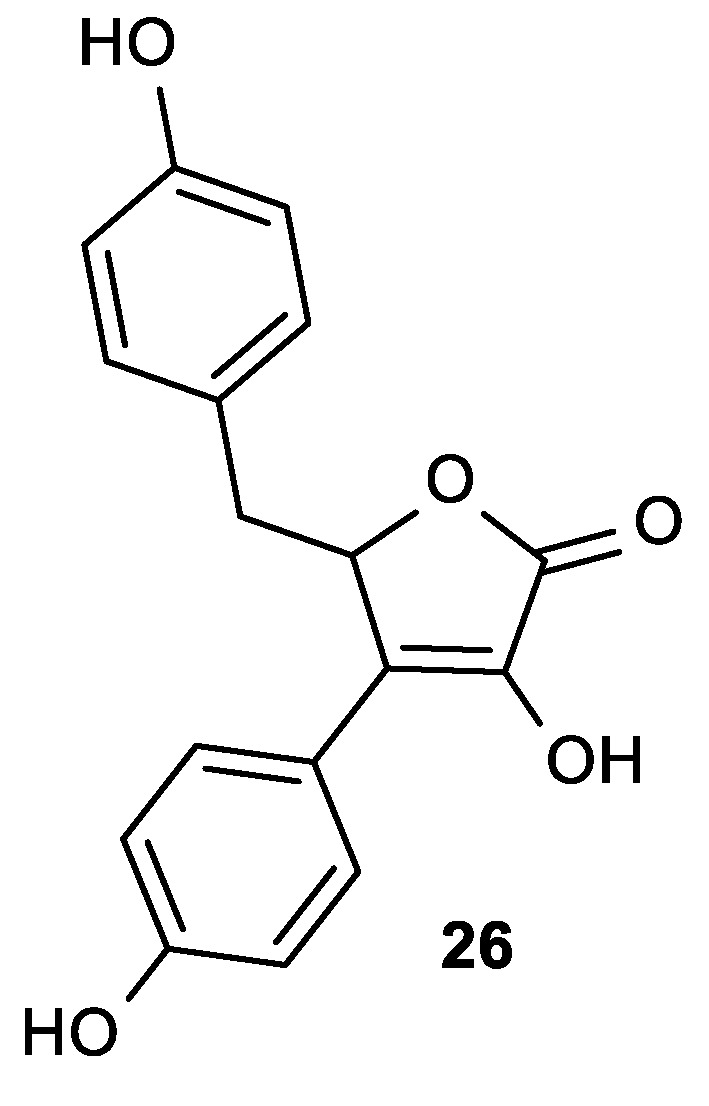
The lipophilic aromatic butenolide derivative **26**.

Cotelle et al. synthesized lipophilic ascorbic acid derivatives with aromatic moieties in positions 3 and 5 that exhibited antioxidant activities close to that of ascorbic acid (Figure 10) [95]. Compound **26** was shown to be a powerful inhibitor of the Cu^2+^ or 2,2′-azobis-(2-amidinopropane)dihydrochloride (AAPH), which induced oxidation of human low-density lipoproteins (LDL) (Table 6).

**Figure 11:**
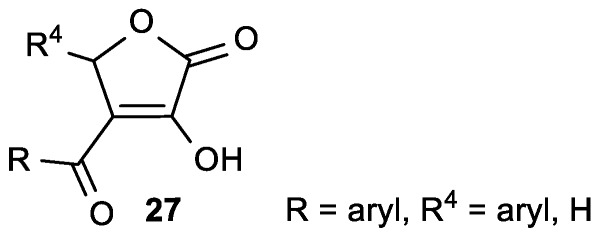
3-Benzoyl-substituted butenolide derivatives.

Moreover, Weber et al. prepared ascorbic acid analogues with a benzoyl moiety at position 3 of the lactone ring, containing hydroxyl and methoxy substituents at the ortho, para, and meta positions of the aryl moiety (Figure 11) [64]. 3-Benzoyl derivatives **27a** and **c** exhibited more than 50% inhibition in 1,1-diphenyl-2-picrylhydrazyl (DPPH) assay, while 93% inhibition was observed for ASA. Compounds **27b** and **d** exhibited potent superoxide anion scavenging activity, whereas ASA was almost inactive (Table 7). 

**Figure 12:**
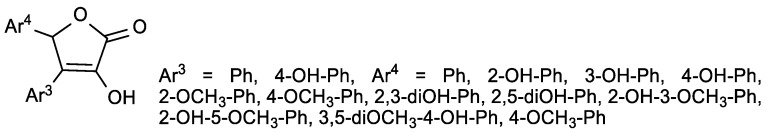
3,4-Diaryl butenolide derivatives as potential antioxidant and anti-inflammatory agents.

Additionally, Weber et al. synthesized ascorbic acid derivatives **28** having aromatic substituents directly linked in the positions 3 and 4 of the lactone ring, and studied the effect of phenol moieties on biological activity (Figure 12) [96]. Compound **28a**, bearing a 2,3-dihydroxy phenyl ring in the position 4 of the lactone, appeared to be the most powerful antioxidant with the best DPPH scavenging activity (IC_50_ = 10.3 µM), superoxide anion quenching capacity (IC_50_ = 0.187 µM), and lipid peroxidation inhibitory effect (IC_50_ = 0.129 µM) (Table 8). Since oxygen-derived free radicals are known to contribute to inflammatory disorders, the inflammation effect of the diaryl compounds **28** was investigated, showing their anti-inflammatory activity, compared with that of indomethacin and ketorolac, and cyclooxygenase inhibition [96]. 

## 3. Antitumor and Antiviral Activities of l-Ascorbic Acid Derivatives

As already mentioned, intravenous administration of a high-dose of ASA, that increases plasma ASA level compared to concentration achieved by oral administration, has been successfully applied for the treatment of cancer patients [18,97,98]. With the aim of solving the instability of ASA, many ASA derivatives have been synthesized—such as 2-*O*-α-d-glucopyranosyl-l-ascorbic acid (ASA-2G, **15**, Figure 7)—which have significantly inhibited tumor growth in tumor-bearing mice [99]. The antitumor activity of ASA-2G (**15**) was caused by ROS, generated by ASA, and released by hydrolysis of ASA-2G. Therefore, ASA-2G could be an alternative drug in intravenous high-dose ASA therapy to improve the instability of ASA. Among two types of lipophilic derivatives of ASA-2G, having a straight-acyl chain (6-sAcyl-ASA-2G, **16a**–**h**, Figure 7) and a branched-acyl chain (6-bAcyl-ASA-2G, **17a**–**f**, Figure 7) [88,89,100], the antiproliferative activity of 6-bOcta-ASA-2G (**17b**) was shown to have a different mechanism of action than ASA, due to both reduced and oxidized 6-bOcta-ASA that is formed from 6-bOcta-ASA-2G [100]. Similarly to ASA, its stable analogue 2-*O*-*β*-d-glucopyranosyl-l-ascorbic acid (ASA-2*β*-G, **22**, Figure 8) selectively induced cell death, inhibiting the proliferation of cervical carcinoma (HeLa) by the mechanism of cell apoptosis, and cell cycle arrest through stabilization of p53 protein [101]. 

It was found that supplementation with ASA-2P (**20**, Figure 7) effectively reduced the ability of HIF-1α to promote malignant progression in melanoma cells [53]. Besides, ASA-2P (**20**) showed antiviral activity against several human cytomegalovirus (CMV) strains in human foreskin fibroblasts (HFF) and endothelial cells (EC) [102].

### 3.1. Lipophilic ASA Derivatives with Modified Hydroxyl Groups

**Figure 13:**
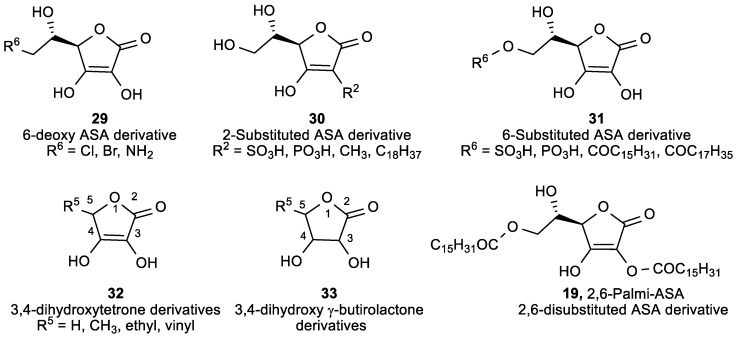
Lipophilic ASA derivatives with modified hydroxyl groups.

Roomi et al. tested the growth suppression of malignant leukemia cell line (P388D1) in vitro by ASA and its isomers—d-ascorbic acid (D-ASA), d-isoascorbic acid (D-IASA), l-isoascorbic acid (L-IASA), and l-dehydroascorbic acid (L-DHASA)—as well as differently substituted ASA derivatives (Figure 13, Table 9) [103]. 

The stereoisomers D-ASA, D-IASA, and L-DHASA had activities similar to that of ASA, which suggests that the cytotoxic effect of ascorbate was not related to the metabolic or ASA activities at the cellular level. Antitumor evaluations displayed similar inhibitory effects for 6-substituted (**31**) and 6-deoxy derivative (**29**) as that of ASA (Figure 13, Table 9), while 2-substituted (**30**) and 2,6-disubstituted ASA (**19**) derivatives did not exhibit activity. Interestingly, dihydroxy *γ*-crotonolactone derivatives (**32**), with or without substituents at the C-5, exhibited cytotoxicity that enhanced with the increasing number of C atoms in the chain at the C-5, while dihydroxy *γ*-butirolactone derivatives (**33**) showed no activity. The results suggest that the dihydroxy *γ*-crotonolactone moiety was crucial for the inhibitory activity, whereas the ethylene glycol residue had no impact on activity. 

Furthermore, the substitution on the C-6 of ASA improved antitumor activity in comparison with ASA. Thus, 6-bromo- [104], 6-chloro [105], 6-amino-, and *N,N*-dimethyl-6-amino-6-deoxy-l-ascorbic acid (Figure S4) [106,107] inhibited the growth of cervical carcinoma (HeLa), laryngeal carcinoma (Hep2), and pancreatic carcinoma (MiaPaCa2). 

**Figure 14:**
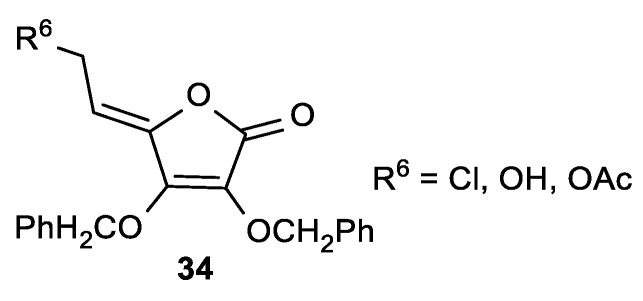
The 6-chloro-2,3-di-*O*-benzyl-l-ascorbic acid derivative.

Among other 6-substituted ASA derivatives (**34**), 6-chloro ASA (**34a**) showed moderate cytostatic activity (IC_50_ ~18 µM) against malignant tumor cell lines HeLa, MiaPaCa-2, Hep2, MCF-7, and SW 620 (Figure 14, Table 10) [108].

**Figure 15:**
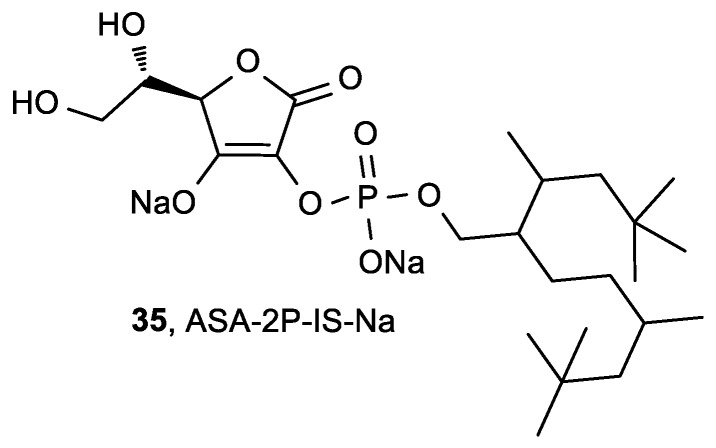
ASA-2P-IS-Na with an inhibitory effect on melanogenesis.

Matsuda et al. reported that disodium isostearyl 2-*O*-l-ascorbyl phosphate (ASA-2P-IS-Na, **35**, Figure 15) inhibited melanogenesis in cultured human melanoma cells, normal human melanocytes, and three-dimensional human skin models to a greater degree than ASA and sodium-2-*O*-l-ascorbyl phosphate (ASA-2P-Na) [109].

Additionally, ASA-2P-IS-Na (**35**) significantly suppressed the cellular tyrosinase activity of cultured human melanoma cells and normal human melanocytes, and showed a small inhibitory effect on matrix metalloproteinase-1 (MMP-1) in normal human fibroblasts, whereas ASA did not inhibit MMP-1 [110]. ASA-2P-IS-Na also exerted collagen synthesis-promoting activity after convertion to ASA by phosphatase.

**Figure 16:**
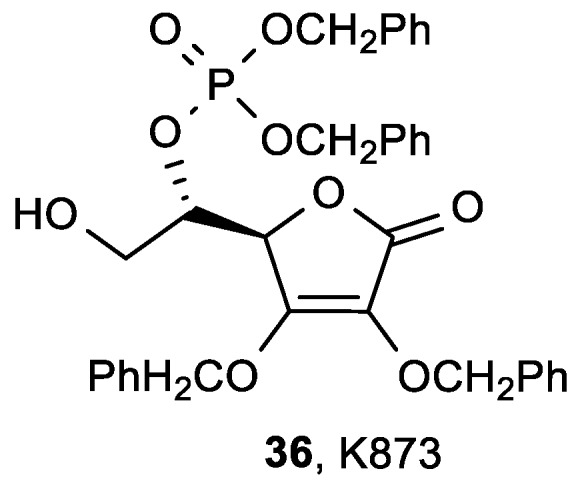
The 5-phosphate derivative K873.

Bordignon et al. found that the 5-phosphate derivative of 2,3-di-*O*-benzyl-l-ascorbic acid (K873, **36**) had an antiproliferative effect superior to ASA on hepatoma (HuH7), colon carcinoma (HT29), Burkitt lymphoma (Raji), and myeloma (CCL155) cell lines (Figure 16, Table 11) [111], and no cytotoxicity to non-neoplastic human cells (human primary hepatocytes).

The compound K873 was also tested in vivo in immunodeficient mice (BALB/c Nude), xenografted with HT29 and PC3 tumor cell lines. The treatment of mice with the 5-phosphate derivative K873 resulted in inhibition of tumor progression. The results suggest that K873 has a similar mechanism of action as ASA, and reduces the expression of genes that are involved in the cell cycle progression. As opposed to ASA, K873 did not enter the cell by the membrane protein SVCT2. The overall results indicate that K873, alone or in a combinatorial therapy, could be a promising anticancer drug. 

3-*O*-Ethylascorbic acid (Figure S5) effectively inhibited the induction of nephroblastomas, an uncommon tumor mostly found in childhood in humans [112], and reduced rat mammary tumor induction [112,113].

Brinkevich et al. studied the relationship between the radical-inhibiting properties and antiviral activity of ASA derivatives, with modified hydroxyl groups 5,6-di-*O*-isopropylidene-2,3-di-*O*-methyl-l-ascorbic acid (Figure S1), 2,3-di-*O*-methyl-l-ascorbic acids (Figure S1), 2-*O*-*α*-d-glucopyranosyl-l-ascorbic (ASA-2G, **15**, Figure 7), 6-*O*-palmitoyl-2-*O*-glucopyranosyl-l-ascorbic (6-sPalmi-ASA-2G, **16g**, Figure 7), and 6-*O*-palmitoyl-l-ascorbic (6-Palmi-ASA, **18**, Figure 7) [114]. It was found that 5,6-di-*O*-isopropylidene-2,3-di-*O*-methyl-l-ascorbic acid, 2,3-di-*O*-methyl-l-ascorbic acids (Figure S1), and compound **18**, as well as 6-*O*-acylated ASA derivative, did not show antiviral activity on the replication of herpes simplex virus type I (HSV-1) in human rhabdomyosarcoma (RD) cell culture, whereas **15** and **16g** proved to be effective inhibitors of HSV-1 replication (Figure 7, Table 12). 

The lower value of EC_50_ and slightly higher toxicity of 6-sPalmi-ASA-2G (**16g**) than those of ASA-2G (**15**) are probably due to higher lipophilicity caused by the 6-palmitoyl side chain in **16g**. 

### 3.2. Conjugates of ASA Derivative and Pyrimidine and Purine Base

**Figure 17:**
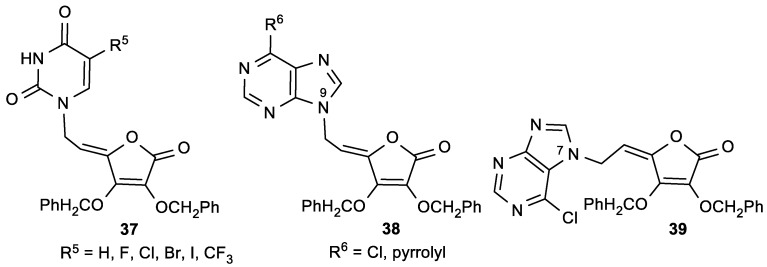
Pyrimidine and purine derivatives of l-ascorbic acid.

Raić-Malić et al. prepared a new class of compounds containing pyrimidine or purine moieties connected via an acyclic unsaturated chain to 2,3-di-*O*-benzyl-4,5-didehydro-5,6-dideoxy-l-ascorbic acid with interesting biological properties (Figure 17, Table 13 and Table 14) [115]. 

Compound **37f**, with a trifluoromethyl substituent at C-5 of uracil, showed a significant antitumor activity—particularly against human T-lymphocytes (Molt4/C8) (IC_50_ = 0.9 µM) (Table 13). The *N*-9-substituted 6-chloropurine regioisomer **38a** showed slight antiproliferative activity against malignant murine and human cells, whereas its *N*-7 isomer **39** displayed a more pronounced inhibition on the majority of the examined cell lines. The pyrimidine derivative **37f** showed the most potent antiviral activities against varicella-zoster virus (TK^+^VZV and TK^−^VZV) and cytomegalovirus (CMV), but at concentrations that were only slightly lower than the cytotoxic concentrations (Table 14). Also, compound **37f** exhibited more potent activity against TK^+^VZV/YS and TK^−^VZV strains than acyclovir (ACV), and greater activity against cytomegalovirus than 9-[(1,3-dihydroxy-2-propoxy)methyl]guanine (DHPG). Moreover, the purine *N*-7 isomer **39** was more active against TK^−^VZV (YS/R) than ACV and (*E*)-5-(2-bromovinyl)-2′-deoxyuridine (BVDU), while the *N*-9 derivative **38a** did not show any appreciable activity.

**Figure 18:**
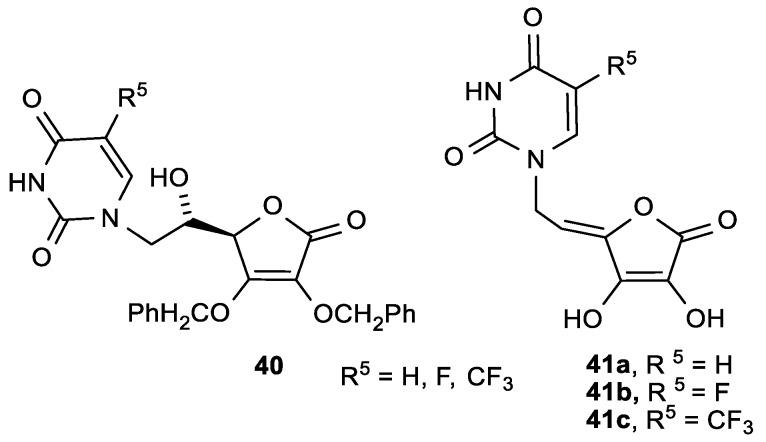
Pyrimidine derivatives of 2,3-di-*O*-benzyl-6-deoxy-l-ASA (**40**) and 4,5-didehydro-5,6-dideoxy-l-ASA (**41**).

Furthermore, in the series of pyrimidine derivatives of 2,3-di-*O*-benzyl-6-deoxy-l-ascorbic acid (**40**) and 4,5-didehydro-l-ascorbic acid (**41**), the compound **41b**—with a 5-fluoro-substituted uracil—showed the best antitumor activities against murine leukemia (L1210/0) (IC_50_ = 1.4 µg/mL) and murine mammary carcinoma (FM3A/0) (IC_50_ = 0.78 µg/mL) (Figure 18, Table 15) [116]. 

**Figure 19:**
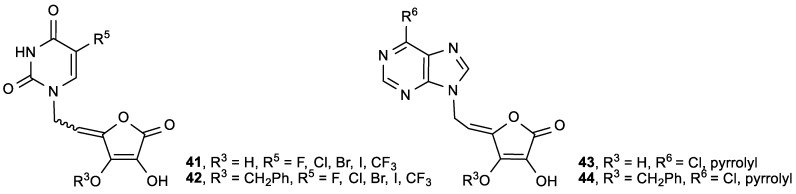
Pyrimidine and purine derivatives of ASA.

The ASA derivatives **40** showed 10-fold less pronounced cytostatic activity on the examined tumor cells in comparison with the unsaturated derivatives **37a**, **b**, and **f**. It was found that among C-5 substituted pyrimidine and purine ASA derivatives with free hydroxyl groups at the C-2 or/and C-3 of the lactone ring, derivatives **41b** showed the most pronounced antitumor activities (Figure 19, Table 16). The compound **41c**, containing a 5-(trifluoromethyl)uracil, induced apoptosis in SW 620 and MiaPaCa-2 cells, and influenced the cell cycle by increasing the cell population in the G2/M phase [117].

**Figure 20:**
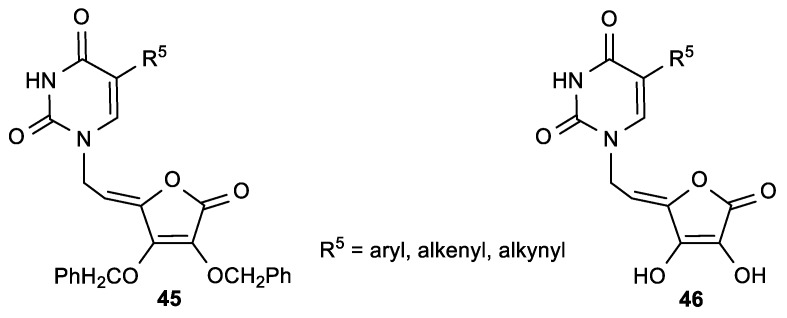
The C-5 aryl-, alkenyl-, and alkynyl-substituted uracil derivatives of ASA.

The purine ASA derivative **44a**, with a 6-chloropurine ring, had inhibitory effects against HeLa (IC_50_ = 6.8 µM) and MiaPaCa-2 cells (IC_50_ = 6.5 µM) (Figure 19, Table 16). The comparison of the cytostatic activities of **37** and **38** (Figure 17, Table 13) with their 2,3-dihydroxy analogues (**41** and **43**, Figure 19, Table 16) showed that the majority of 2,3-di-*O*-benzyl ASA derivatives had better inhibitory effects than their 2,3-dihydroxy analogues. Furthermore, from the series of C-5 aryl, alkenyl and alkynyl substituted uracil derivatives of ASA synthesized by Gazivoda et al. (Figure 20) [118], the 5-propynyluracil ASA derivative (**45a**) exhibited the most pronounced cytostatic activities against all examined tumor cells (IC_50_ = 0.2–0.78 µM). 2,3-Di-*O*-benzyl ASA derivatives, 5-furyluracil (**45b**), 5-vinyluracil (**45c**), 5-ethynyluracil (**45d**), and 5-isopentenyluracil (**45e**), as well as 5-(phenylethynyl)uracil-2,3-dihydroxy-ASA (**46a**), exhibited marked antiproliferative effects against all tumor cell lines, but also cytotoxic activity on normal human fibroblasts (WI 38) (Table 17).

ASA derivative **45a** also exhibited some not highly specific inhibitory activity against vesicular stomatitis virus, Coxsackie B4 virus, and Sindbis viruses (EC_50_ = 1.6 µM) (Table 18). 

**Figure 21:**
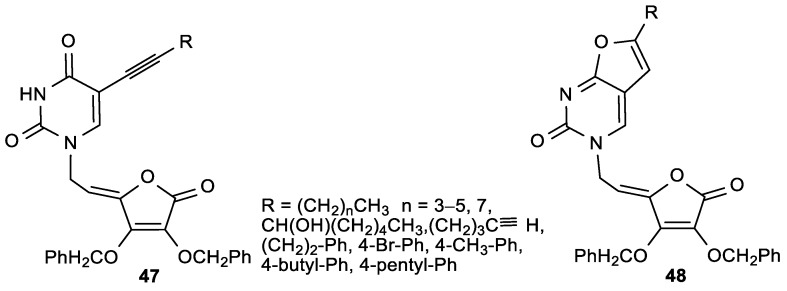
The C-5 substituted pyrimidine and 6-alkylfuro [2,3-*d*] pyrimidine-4,5-didehydro-l-ASA derivatives.

Antiproliferative evaluations of the C-5 alkynyl-substituted pyrimidine ASA derivatives (**47**), prepared by Sonogashira cross-coupling of unsaturated 5-iodouracil of ASA with terminal alkynes, showed that the 5-octinyl-substituted uracil ASA (**47a**, R = hexyl) had the best cytostatic effects against all examined tumor cell lines (IC_50_ = 2–12 µM) (Figure 21, Table 19) [119]. 

The correlation of the antitumoral activity and C-5 alkynyl side chains revealed that the cytostatic activity was dependent on the side chain length. The C-5 alkynyl pyrimidine ASA derivatives (**47**) displayed a better antiviral activity than the furo [2,3-*d*] pyrimidine derivatives (**48**). Compounds **47a** and **c** showed moderate activity against CMV (Davis strain) with EC_50_ = 1.8 and 3.8 µM, respectively (Table 20).

The mechanism of antiviral action of ASA derivatives should be different from that of acyclic nucleoside analogues, such as ganciclovir, due to the lack of free hydroxyl group in ASA moiety, which excluded that these compounds can be phosphorylated.

**Figure 22:**
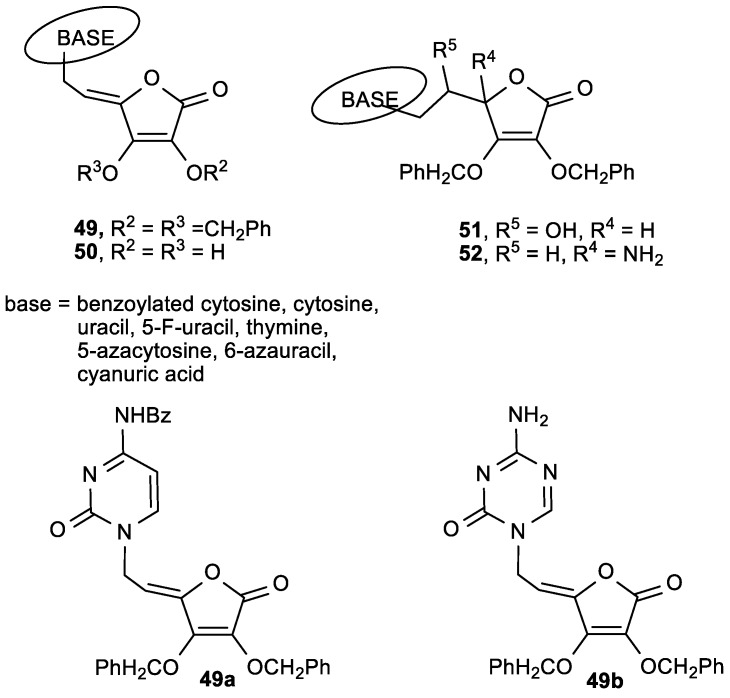
Novel 5- or 6-azapyrimidine and cyanuric acid ASA derivatives.

The cytostatic activity evaluation of cytosine, 5-azacytosine, uracil, 6-azauracil, and cyanuric acid ASA derivatives prepared by Wittine et al. indicates that compounds did not show antiproliferative effects on tested cell lines (Figure 22) [120]. However, unsaturated cytosine ASA derivative (**49a**), with a double bond conjugated with the lactone ring, showed marked inhibitory activity on metastatic breast epithelial carcinoma (MCF-7), HepG2, and HeLa cell lines at micromolar concentrations, but also exerted a strong cytostatic effect on WI 38 (Table 21). Potent antitumor activity against tumor cell lines, with IC_50_ values ranging from 0.92 to 5.91 µM, was also observed for the 5-azacytosine ASA derivative (**49b**). The flow cytometric analysis of the cell cycle revealed that the compound **49b** triggers S phase arrest, which indicates its influence on DNA replication.

**Figure 23:**
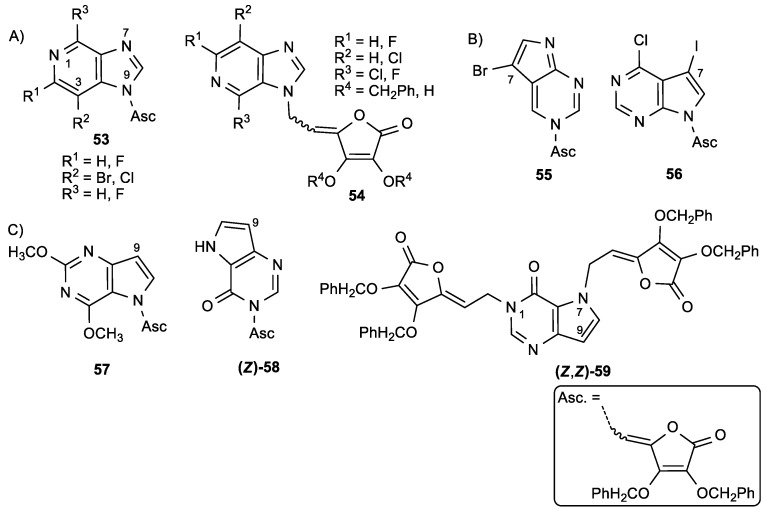
(**A**) 3-Deazapurine derivatives of ASA, (**B**) 7-deazapurine derivatives of ASA, and (**C**) 9-deazapurine derivatives of ASA.

Stipković Babić et al. synthesized conjugates of halogenated 3-, 7-, and 9-deazapurine and ASA in which the lactone ring was connected via ethylidene linker to the corresponding deazapurines. The antiproliferative activity of conjugates was tested against human malignant tumor cell lines, as well as normal murine fibroblasts (3T3) (Figure 23, Table 22) [121]. The results showed that the 2,6-dimethoxy-9-deazapurine and ASA conjugate **57** had the strongest antiproliferative activity on CEM/0 (IC_50_ = 4.1 µM) and L1210/0 (IC_50_ = 4.7 µM) cells (Table 22). Furthermore, 9-deazahypoxanthine derivative disubstituted with ASA, (*Z*, *Z*)-**59**, showed the most potent inhibitory activity on HeLa (IC_50_ = 5.6 mM) and L1210/0 cells (IC_50_ = 4.5 µM). The antiviral activity showed that the 2,6-difluoro-3-deazapurine ASA derivative **54a** had the most potent activity against human cytomegalovirus (HCMV) (AD-169 and Davis) that was similar to the activity of ganciclovir, a well-known anti-HCMV drug (Table 23). Additionally, compound **54a** did not inhibit the growth of normal human embryonal lung (HEL) cells.

**Figure 24:**
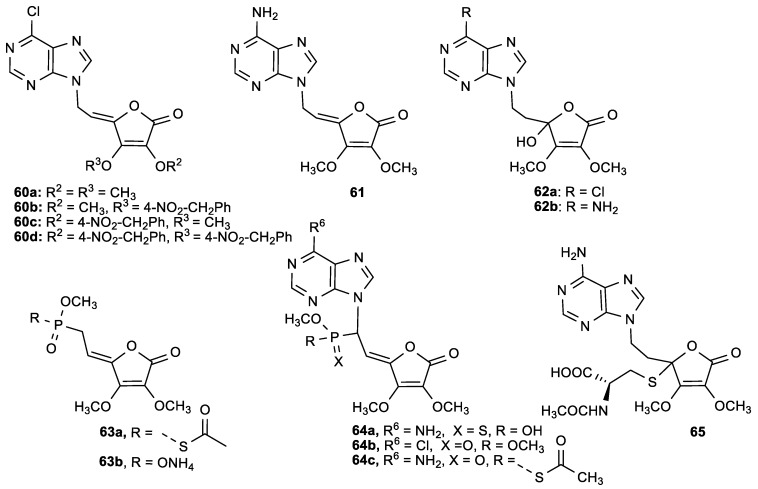
Conjugates of purines and butenolides (**60**−**62**), phosphonobutenolides (**63**, **64**) and the *N*-acetyl-l-cysteine adduct (**65**).

Hakimelahi et al. performed antitumor and antiviral evaluations of a series of purine and butenolide conjugates (Figure 24, Table 24) [122,123]. In order to understand the mechanism of their biological activity, compounds were tested for their inhibitory activity toward *S*-adenosyl-l-homocysteine (AdoHcy) hydrolase and ribonucleotide diphosphate reductase (RDPR)—enzymes that are important in the metabolic pathways in cell division—showing that the activity is connected with the reaction of the C4=C5 double bond with nucleophilic parts of the protein, like l-cysteine (Table 25). The 6-chloropurine butenolides **60a**–**c** showed significant antiviral activity against varicella-zoster virus and anticancer activity, but were weak inhibitors of AdoHcy hydrolase (Table 24). The adenine butenolide conjugate **61** showed a specific antiproliferative effect on leukemia (P388) cells and inactivated AdoHcy hydrolase. On the other hand, its saturated derivatives **62b** and **65** did not show any antiproliferative effects, indicating that the double bond is important for biological activity. Phosphonobutenolides **63**, without a purine base, did not exhibit anticancer nor antiviral activity and inhibitory effect of RDPR, which confirmed that the purine base was crucial for the interaction with the target protein.

**Figure 25:**
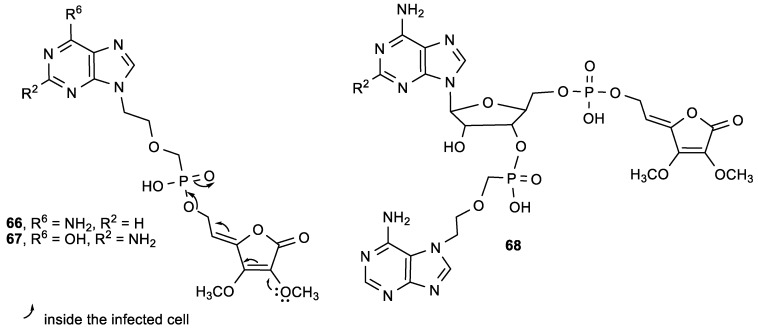
The butenolide derivatives of 9-[2(phosphonomethoxy)ethyl]adenine (PMEA) (**66**) and 9-[2(phosphonomethoxy)ethyl]guanine (PMEG) (**67**) and the nucleotide containing butenolide **68**.

Having in mind that low lipophilicity of nucleotide analogues is connected with their difficulties in penetrating the cell membrane and entering the cell, Hakimelahi et al. prepared lipophilic prodrugs (**66**–**68**) of known antiviral compounds (9-[2(phosphonomethoxy)ethyl]adenine (PMEA) and 9-[2(phosphonomethoxy)ethyl]guanine (PMEG)) by adding a butenolide ester into the molecule, hypothesizing that this modification would result in an increase of antiviral activity compared to their parent molecules (Figure 25) [124]. ASA analogues **66**–**68** showed better antiviral activities in vitro against human immunodeficiency viruses (HIV-1, HIV-2) and Moloney murine sarcoma virus (MSV) than their parent molecules (Table 26). Compounds **66** and **68** also inhibited HSV-1 virus in vivo in mice. The adenine butenolide **66** efficiently reduced the formation of tumors induced by MSV in mice, and increased the survival time of mice infected with MSV. The authors presumed that inside the infected cells, the oxygen at position 2 of the butenolide facilitates the hydrolysis of the ester, releasing the active part of the molecule (Figure 25).

### 3.3. Conjugates of ASA Derivative and Triazole and Imidazole Moiety

**Figure 26:**
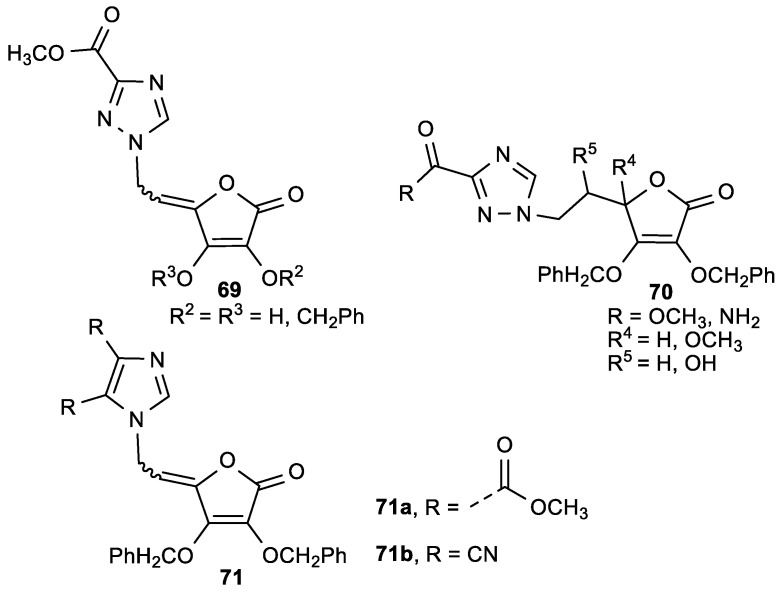
The 1,2,4-triazole (**69**, **70**) and 4,5-disubstituted-imidazole (**71**) ASA derivatives.

The 1,2,4-triazole and imidazole ASA derivatives (**69**–**71**) were prepared and evaluated for their inhibitory activity against the hepatitis C virus (HCV) replication and human tumor cell proliferation (Figure 26, Table 27) [125].

ASA derivative **71a** with a 4,5-disubstituted carboxymethyl, and **71b**, with a dicyano imidazole moiety linked with an ethylidene spacer to a lactone ring, exhibited cytostatic activities against all tested tumor cells, and were selective for human T-cell acute lymphoblastic leukemia cells (CEM/0) (Table 27). Imidazole ASA derivative **71a** might act as inhibitor of inosine monophosphate dehydrogenase (IMPDH) activity that is known as enzyme of de novo purine nucleotide biosynthesis. This compound has also been shown to inhibit replication of the hepatitis C virus.

Having in mind the biological potential of the 1,2,3-triazole moiety, a series of novel 1,2,3-triazolyl appended *γ*-butenolides (Figure S6) were synthesized from ASA and screened for anticancer activity. However, none of the novel butenolides showed significant antiproliferative activity with IC_50_ <20 μM.

### 3.4. Conjugates of ASA Derivative and Triterpene

**Figure 27:**
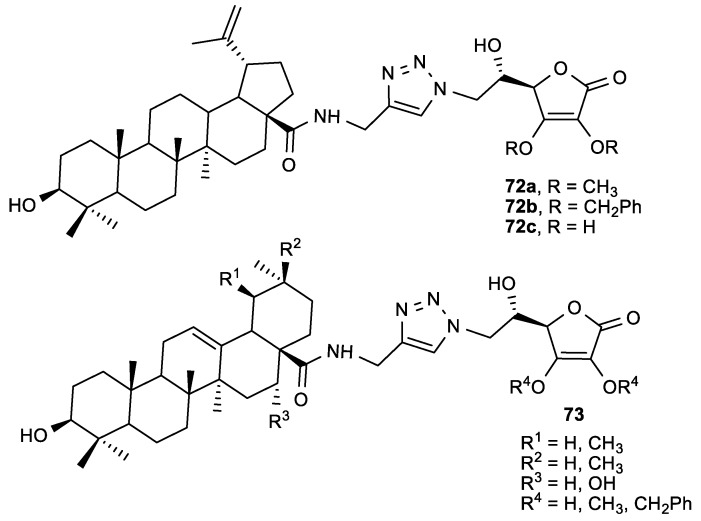
The pentacyclic triterpene ASA conjugates.

Considering the potential synergistic effect of pentacyclic triterpenes and ASA, Wang et al. prepared molecular hybrids consisting of echinocystic acid (EA), oleanolic acid (OA), ursolic acid (UA), and betulinic acid (BA) connected to ASA via 1,2,3-triazole or an amide linker (Figure 27) [126].

The hybrids were tested for their anti-influenza activity against A/WSN/33 virus (Table 28). Among all tested conjugates, the 2,3-di-*O*-benzyl-ASA derivative (**72b**) had the most potent anti-influenza activity (EC_50_ = 8.7 µM) without being toxic to Madin-Darby Canine Kidney (MDCK) cells. Compound **72b** is a promising influenza virus entry inhibitor that inhibits hemagglutinin (HA) protein, which is important in the attachment of the influenza viruses to the target host cells.

### 3.5. Butenolide Derivatives

**Figure 28:**
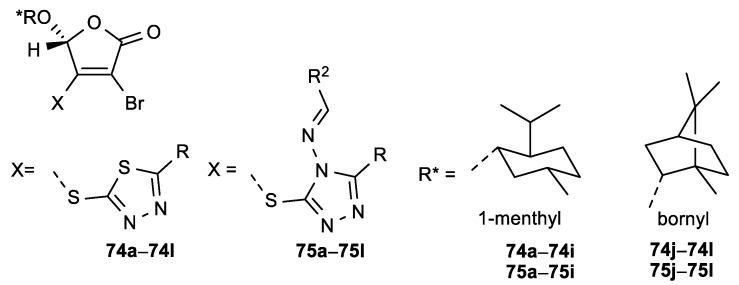
Butenolides substituted with 1,3,4-thiadiazoles (**74a**–**l**) or 1,2,4-triazole Schiff bases (**75a**–**l**) at position C-3 of the lactone ring.

Among butenolide derivatives, two series of molecular hybrids—consisting of chiral 1,3,4-thiadiazoles derivatives or 1,2,4-triazole Schiff bases and *γ*-substituted butenolide—were synthesized, and their antiproliferative activity was tested on HeLa cell line (Figure 28, Table 29) [127,128]. The comparison of activities for these two series showed that compounds bearing the substituted 1,3,4-thiadiazole had overall better anticancer activity than the butenolide hybrids bearing the 1,2,4-triazole Schiff bases. Of all the evaluated compounds, the 1,3,4-thiadiazole derivative **74e** with a 4-nitrophenyl substituent showed the most potent antiproliferative activity (IC_50_ = 0.9 µM) and, among the 1,2,4-triazole Schiff bases, the compound **75l** with a 2-hydroxyphenyl substituent showed the best inhibitory effect (IC_50_ = 1.8 µM)—which was, in both cases, superior to Cisplatin.

**Figure 29:**
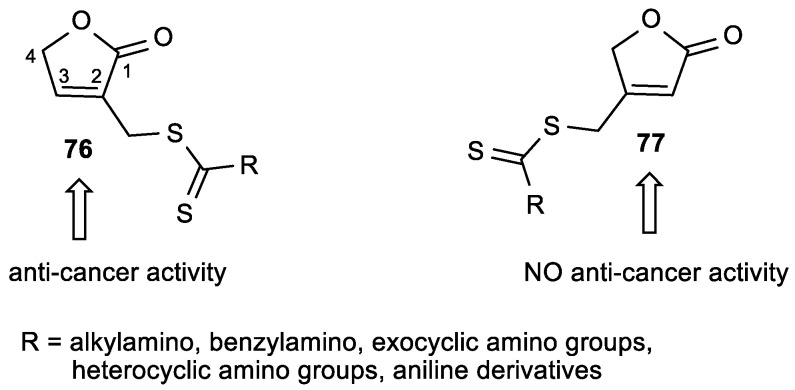
The butenolides **76** and **77** with a dithiocarbamate side chain.

Wang et al. prepared butenolides, with a dithiocarbamate moiety either at position 2 (**76**) or at position 3 (**77**) of the lactone ring, as potential anticancer agents (Figure 29) [129]. The results of the anticancer activity of butenolides **76** and **77** in human cancer cell lines indicate that the position of the side chain is essential for anticancer activity (Table 30). Compounds **76d**–**f**, bearing heterocyclic amines, showed strong antiproliferative effects to HeLa cell line, with IC_50_ in the range of 0.77–2.63 µM, which was superior to 5-fluorouracil.

**Figure 30:**
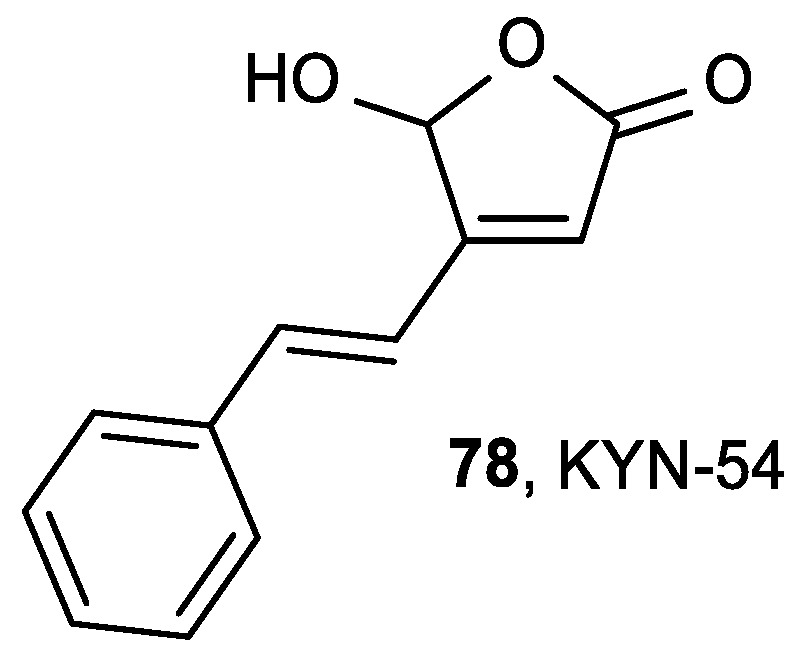
Butenolide KYN-54 (**78**) with antitumor activity.

5-Hydroxy-4-(2-phenyl-(*E*)-ethenyl)-2(5H)-furanone (KYN-54, **78**) is a butenolide derivative with anticancer activity and inhibitory effect to intestinal carcinogenesis in rats (Figure 30) [130].

Mori et al. studied the effect of KYN-54 (**78**) in mice with pulmonary carcinogenesis induced by the carcinogenic agent 4-(methylnitrosamino)-1-(3-pyridyl)-1-butanone (NNK) [131]. KYN-54 did not have a chemopreventive, but rather a promoting, effect on the pulmonary carcinogenesis in mice.

**Figure 31:**
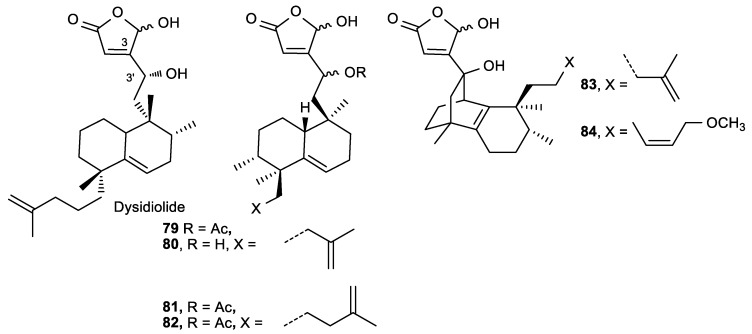
Compounds structurally related to dysidiolide as potential.

Sesterterpenolide derivatives of dysidiolide with a butenolide moiety were synthesized by Marcos et al. with the aim of enhancing the antitumoral effect of dysidiolide (Figure 31) [132].The compounds **79**–**83** had slightly better, or the same, antiproliferative effects as dysidiolide (Table 31). Derivative **83** with a tricyclic framework had the highest activity against leukemia cells HL-60, with an IC_50_ of 0.3 µM. The configuration at C-3′ did not affect antitumoral activity, while the -hydroxybutenolide was crucial for the activity, showing decreased biological activity when it was replaced with a furan. A significant structural modification of the isoprenyl group also led to a decrease of the antiproliferative activity.

Presley et al. isolated a butenolide diterpene from the plant *Metaporana sericosepala* (Figure S7) [133]. The compound exhibited inhibitory activity on ovarian cancer cells (A2780).

**Figure 32:**
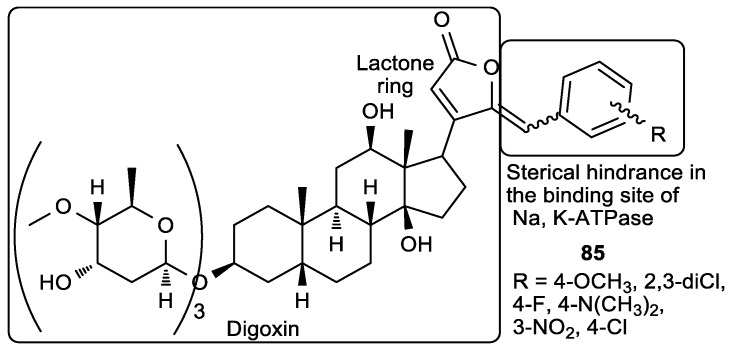
*γ*-Benzylidene derivatives of digoxin.

Alves et al. synthesized digoxin derivatives of butenolide with a benzylidene at C-4 of the lactone ring (Figure 32) [134]. The substituent on the aromatic ring had an influence on the antiproliferative activity, and the 4-(dimethylamino)benzylidene derivative of digoxin (**85a**) showed the most potent activity on HeLa and colon carcinoma (RKO) cells, as well as human lung fibroblasts (WI-26 VA4) (Table 32). However, evaluation of their mechanism of action showed that these compounds do not inhibit the sodium pump and Na- and K-ATPase activity, which was opposite to digoxin.

**Figure 33:**
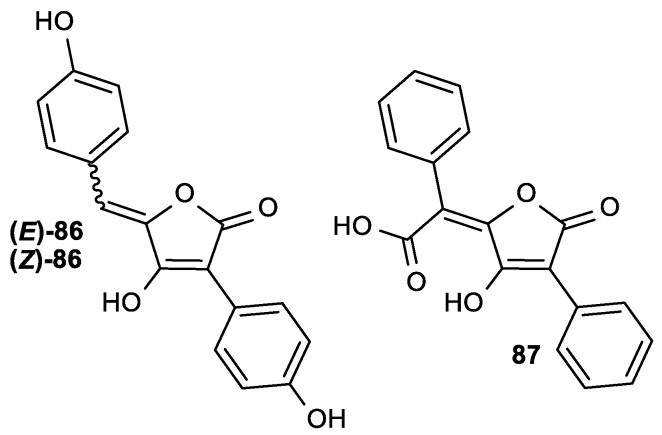
Aspulvinones with a butenolide in their structure: (*E*)-**86**, (*Z*)-**86** and pulvic acid (**87**).

Some aspulvinone derivatives isolated from the fungus *Aspergillus terreus* showed antiviral activity against influenza A H1N1 virus (IC_50_ = 29.1–56.9 µg/mL) without being toxic to A549 and Madin-Darby Canine Kidney (MDCK) cells (Figure 33, Table 33) [135].

Moreover, compound (*E*)-**86** was an inhibitor of the viral neuraminidase (NA), and the comparison of the molecular docking of (*E*)-**86** and (*Z*)-**86** isomers into the binding site of NA showed that the *E*-configuration of the double bond was crucial for the interactions of the molecule with the amino acid residues in the binding site of NA.

## 4. Conclusions

This review focuses on the diverse and potent biological activity of l-ascorbic acid and its derivatives, and is an attempt to provide an outlook on the development of compounds containing l-ascorbic acid or butenolide moiety as antioxidant, anticancer, or antiviral agents.

Vitamin C is a naturally occurring potent antioxidant due to its free radical and reactive oxygen species scavenging action, redox potential, and prevention of oxidative damage to lipids and other macromolecules. Depending on the concentration gradient, it can exhibit bimodal activity as either antioxidant or prooxidant in pathological conditions. In addition to being a general antioxidant, recent advances have identified the role of ascorbate in the epigenetic regulation of gene activity by functioning as a cofactor for Tet enzymes, leading to DNA demethylation. The susceptibility of vitamin C to thermal and oxidative degradation together with its poor liposolubility has led to its structural modifications by the introduction of diverse lipophilic moieties.

Thus, some lipophilic ASA derivatives obtained by modified hydroxyl groups showed an improved antioxidant potential over the ASA itself. For instance, 6-*O*-acyl-ASA derivatives with long alkyl chain and 6-phenolic esters of ASA showed stronger radical scavenging ability in comparison to ASA. It was observed that both a long alkyl chain and the electron donating activity of the enolic 2- and 3-hydroxyl groups are required in the suppression of lipid peroxidation. It appeared that radical scavenging activity of the 3-alkylated ASA derivatives was lower than that of 2-alkylated ASA derivatives and ASA. The long hydrophobic 6-palmitate chain had a crucial role for the improved binding affinity to lipoxygenase 5-LOX of 6-Palmi-ASA, which exhibited a more potent inhibitory effect of 5-LOX than known inhibitors. Although the increasing length of acyl group in the glucoside ASA derivatives series tended to increase the scavenging activity, both straight-acyl chain and branched-acyl chain had lower radical scavenging ability than ASA itself, with no additional effect of conversion of straight- to branched-acyl chain on activity. On the contrary to ASA-2G that exhibited lower scavenging potency than ASA, *β*-glucoside linkage in ASA-2*β*-G had an impact on its improved antioxidant effect. Among aromatic butenolide derivatives, a 2,3-dihydroxy phenyl ring on the C-4 of the lactone moiety had the best influence on scavenging and lipid peroxidation inhibitory effects.

Regarding the antitumor activity of ASA derivatives with modified hydroxyl groups, the 2,3-dihydroxy butenolide moiety was shown to be crucial for antiproliferative effects, whereas the ethylene glycol residue had no impact on activity. However, the 5-phosphate derivative of ASA with benzylated 2,3-dihydoxyl groups exhibited cytostatic effects on hepatoma (HuH7), colon carcinoma (HT29), Burkitt lymphoma (Raji), and myeloma (CCL155) cells superior to ASA, with no toxicity to normal non-neoplastic human cells. Halogen substitution on the C-6 position of ASA was shown to improve antitumor activity in comparison to ASA. A cytostatic effect was not observed when the γ-crotonolactone was reduced to γ-butirolactone ring. Pyrimidine and purine ASA derivatives, in which nucleobase was connected to sugar mimetic butenolide moiety via ethylidene spacer, exhibited pronounced antitumor activities, emphasizing conjugates of 5-(trifluoromethyl)-, 5-fluoro-, and 5-propynyl-substituted uracil and ASA with antiproliferative effects on malignant tumor cell lines in nM range. While 5-(trifluoromethyl)uracil ASA derivative showed some selectivity (selectivity index, SI > 60) in its activity, 5-fluoro- and 5-propynyluracil ASA derivatives exhibited cytotoxic effects on normal human fibroblasts. Other pyrimidine and purine ASA derivatives with inhibitory activities on the growth of malignant tumor cells also exerted cytotoxic effects on normal cells, except for representative of 9-deazapurine ASA conjugate that showed selective antiproliferative activity on leukemia (CEM/0 and L1210/0) cells without being toxic on normal cells. Interestingly, *N*-7 purine regioisomer displayed a more pronounced inhibition on malignant tumor cells than its *N*-9 isomer. Adenine ASA conjugate showed a specific antiproliferative effect on leukemia (P388) cells and inactivated S-adenosyl-l-homocysteine (AdoHcy) hydrolase. Moreover, ASA derivatives with imidazole moiety linked by ethylidene exhibited selective inhibitory effects on human T-cell acute lymphoblastic leukemia cells (CEM/0). Generally, conformationally restricted ethylidene spacer between lactone and nucleobase had profound effects on antitumor activities. Moreover, the majority of 2,3-di-*O*-benzyl ASA nucleobase conjugates had better inhibitory activities than the corresponding 2,3-dihydroxy analogues. Cyclization of the 5-alkynyluracil ASA derivatives to the fused bicyclic furo [2,3-d] pyrimidine ASA derivatives caused a reduction of the antitumor effects. In butenolide substituted series, C-2 position of the dithiocarbamate in butenolide moiety was shown to be essential for anticancer activity, while among 3-substituted 1,3,4-thiadiazole and 1,2,4-triazole Schiff bases, 1,3,4-thiadiazole-substituted butelidene derivatives showed better antiproliferative activity.

Referring to the antiviral potency of ASA derivatives, we can emphasize that 5-(trifluoromethyl)uracil ASA conjugate exhibited more potent activity against varicella-zoster virus (VZV) and cytomegalovirus (CMV) than known antiviral drugs acyclovir and ganciclovir, albeit at concentrations that were only slightly lower than the cytotoxic concentrations. On the contrary, 2,6-difluoro-3-deazapurine ASA derivative showed potent and selective anti-CMV activity that was similar to the activity of ganciclovir, with no cytotoxicity on normal human embryonal lung (HEL) cells. Purine *N*-7 regioisomer was more active against VZV (SI > 4) than acyclovir, while its *N*-9 isomer did not show any appreciable activity, which is in agreement with antitumor activity. Phosphonate derivatives of purine and ASA conjugates as lipophilic prodrugs were shown to be selective in their antiviral activities against human immunodeficiency viruses (HIV-1, HIV-2). Besides, pentacyclic triterpene ASA conjugates with 1,2,3-triazole spacer exhibited anti-influenza A/WSN/33 virus activity, confirming that 2,3-di-*O*-benzylated l-ASA skeleton is crucial for their antiviral potencies, regardless of the triterpene and linker moieties. Additionally, some aspulvinone derivatives containing butenolide skeleton had significant anti-influenza A (H1N1) virus activities showing that the *E*-configuration of the double bond is crucial for the interaction with viral neuraminidase (NA).

We may conclude that the above-mentioned observations may reflect a new perspective on the development of l-ascorbic acid and its derivatives possessing diverse biological properties, and could be beneficial for medicinal chemists and chemical biologists in the search for more potent candidates imparted with potent antioxidant, antiproliferative, or antiviral activities.

## Figures and Tables

**Table 1:** 1,1-Diphenyl-2-picrylhydrazyl (DPPH) assay, the redox potentials and inhibitory effects on lipid peroxidation in rat liver microsomes of 2-RASA and 3-RASA derivatives.

Comp.	LO Inhibition		Scaveng. of DPPH (%)	Peak Potential (mV) ^c^
	(%) ^a^	IC_50_^b^ (μM)		
**9a**, R^2^ = (CH_2_)_17_CH_3_	88		>90	240
**10a**, R^3^ = (CH_2_)_11_CH_3_	88	3.1	61	300
**10b**, R^3^ = CH_2_CO(CH_2_)_9_CH_3_	88	3.3	39	380
ASA			>90	210

^a^ LO = lipid peroxidation, calculated as % inhibition at 1 μM against Fe^3+^-ADP induced lipid peroxidation. ^b^ IC_50_ = dose resulting in 50% inhibition. ^c^ at pH 7.4.

**Table 2:** The radical scavenging activity and cytotoxicity of 6-*O*-acyl-l-ascorbic acid-3-*O*-phosphates on 95-D cells.

Comp.	Cytotoxicity	Radical Scaveng. Activity	
	IC_50_ (×10^−5^ M)	EC_50_ (×10^−5^ M)	IC_50_/EC_50_
**11a**, 6-Laur-ASA-3P	8.8	7.6	1.16
**11b**, 6- Myri-ASA-3P	5.3	6.5	0.82
**11c**, 6-Palmi-ASA-3P	4.7	6.9	0.68
**11d**, 6-Stear-ASA-3P	4.4	7.3	0.60

**Table 3:** The antioxidant properties of ASA phenolic esters determined by the DPPH assay.

Comp.	Scaveng. of DPPH
	EC_50_ (μM)
**12**, 6-Prot-ASA	0.08
**13**, 6-Gal-ASA	0.11
**14**, 6-Caf-ASA	0.20
ASA	0.42
Protocatechuic acid	0.17
Gallic acid	0.10
Caffeic acid	0.17

**Table 4:** The radical scavenging activity of glucoside ASA derivatives.

Comp.	Scaveng. of DPPH	
	(%)	EC_50_ (×10^−5^ M)
**15**, ASA-2G	68.9	4.5
**16**, 6-sAcyl-ASA-2G	69.2–77.5	4.1–5.9
**17**, 6-bAcyl-ASA-2G		3.1–3.7
**18**, 6-Palmi-ASA	95.7	2.9
**19**, 2,6-Palmi-ASA	23.7	
**20**, ASA-2P	1.5	
**21**, ASA-2S	0.0	
ASA	94.3	2.4
α-Tocopherol	90.9	1.9

**Table 5:** The antioxidant activity of vitamin C and E conjugates determined by the lipid peroxidation assay.

Comp.	LO ^a^ Inhibition IC_50_ (μM)
**23**	12
**24a**, **24b**	12
**24c**, **24d**	7
ASA	14
α-tocopherol	37

^a^ LO = lipid peroxidation.

**Table 6:** Antioxidant activity of the aromatic butenolide **26**.

Comp.	EC_50_/μM		LO ^a^ Inhib.	
	OH Inhib.	DPPH Inhib.	Cu^2+ b^/μM	AAPH ^b^/μM
**26**	2	16	0.1	0.18
ASA	30	13	2.5	-

^a^ LO = lipid peroxidation. ^b^ LO induced by Cu^2+^ or AAPH.

**Table 7:** The DPPH and superoxide anion scavenging activities of 3-benzoyl-substituted butenolides.

Comp.	Scaveng. of DPPH (%)	Scaveng. of Superoxide Anion
**27a**, R = 2-OCH_3_-Ph, R^4^ = H	55.0	58% = IC_50_ = 3.63 × 10^−3^ M
**27b**, R = 3-OH-Ph, R^4^ = H	33.2	91% = IC_50_ = 1.45 × 10^−3^ M
**27c**, R = 2-OH-Ph, R^4^ = H	51.1	NT ^a^
**27d**, R = 3-OH-Ph, R^4^ = 2-OH-Ph	47.7	89% = IC_50_ = 1.35 × 10^−3^ M
ASA	93.3	24%

^a^ NT = not tested.

**Table 8:** The antioxidant activity of 3,4-diaryl butenolide.

Comp.	Scaveng. of DPPH IC_50_ (μM)	Scaveng. of Superoxide Anion IC_50_ (μM)	LO ^a^ Inhibit. IC_50_ (μM)
**28a**, Ar^3^ = Ph, Ar^4^ = 2,3-diOH-Ph	10.3	0.187	0.129
ASA	46.6	24	Prooxidant
Vitamin E	19.6	NT ^b^	NT ^b^

^a^ LO = lipid peroxidation. ^b^ NT = not tested.

**Table 9:** The inhibitory effect of ASA and its derivatives on the growth of leukemia cells.

Comp.	EC_50_ (µg/mL) P388D1
Ascorbic acid and isomers	3
**18**, 6-Palmi-ASA	2
**31a**, ASA-6-stearate (6-Stear-ASA)	2.5
**29a**, 6-Cl-ASA	1
**29b**, 6-Br-ASA	1
**32a**, 3,4-dihydroxytetrone, R^5^ = H	3
**32b**, 3,4-dihydroxy-5-methyltetrone, R^5^ = CH_3_	2
**32c**, 3,4-dihydroxy-5-ethyltetrone, R^5^ = ethyl	0.6
**32d**, 3,4-dihydroxy-5-vinylltetrone, R^5^ = vinyl	0.35

**Table 10:** The cytostatic effects of the 6-chloro-2,3-di-*O*-benzyl-l-ASA derivative **34a**.

Comp.	IC_50_ (μM)					
	HeLa	MCF-7	MiaPaCa-2	Hep2	SW620	WI38
**34a**, R^6^ = Cl	17	17	18	17	19	26

**Table 11:** The antiproliferative activities of the 5-phosphate derivative K873.

	IC_50_ (mM)	
	ASA	36, K873
HuH7	1.0	0.1
HT29	1.5	0.15
Raji	1.3	0.086
CCL155	2.0	0.1

**Table 12:** The anti-herpes simplex virus type I (HSV-1) activity of the glucoside ASA derivatives.

Comp.	EC_50_ (μM) HSV-1	MNTC ^a^ (μM)
**15**	16.3	1183
**16g**	4.5	86.81
ASA	>2270	2270

^a^ MNTC = maximum non-toxic concentration.

**Table 13:** The cytostatic effects of the pyrimidine and purine l-ASA derivatives.

Comp.	IC_50_ (μM)				
	L1210/0	FM3A/0	Molt4/C8	CEM/0	Hef522
**37a**, R^5^ = H	12.9	17.7	4.8	13.0	50
**37b**, R^5^ = F	5.1	3.4	10.9	15.1	40
**37c**, R^5^ = Cl	12.2	16.9	6.1	6.0	30
**37d**, R^5^ = Br	7.5	17.1	3.9	3.5	30
**37e**, R^5^ = I	7.5	22.6	3.9	3.3	40
**37f**, R^5^ = CF_3_	2.0	3.6	0.9	1.6	60
**38a**, R^6^ = Cl	11.6	16.4	14.8	15.9	50
**38b**, R^6^ = pyrrolyl	110	>200	3.9	5.8	>100
**39**	4.1	11.4	6.8	4.4	20

**Table 14:** The antiviral activities of the pyrimidine and purine l-ASA derivatives.

Comp.	EC_50_ (μM)						CC_50_ (μM)
	TK^+^VZV		TK^−^VZV		CMV		
	YS	OKA	07/1	YS/R	AD-169	Davis	
**37f**, R^5^ = CF_3_	0.5	0.3	0.5	0.3	>0.5	0.4	1
**38b**, R^6^ = pyrrolyl	1.5	1.2	2.8	0.6	>0.5	>0.5	4
**39**	>5	5.0	>5	3.8	>5	>5	20
ACV	0.78	0.16	17	12	NT ^a^	NT ^a^	>100
BVDU	0.005	0.001	>50	>50	NT ^a^	NT ^a^	>100
DHPG	NT ^a^	NT ^a^	NT ^a^	NT ^a^	1	5	>50
HPMPC	NT ^a^	NT ^a^	NT ^a^	NT ^a^	0.11	1	NT ^a^

^a^ NT = Not tested.

**Table 15:** The antiproliferative effect of the pyrimidine 4,5-didehydro-5,6-dideoxy-l-ASA derivative.

	IC_50_ (μg/mL)			
	L1210/0	FM3A/0	Molt4/C8	CEM/0
**41b**, R^5^ = F	1.4	0.78	31.8	20.9
5-FU	0.04	0.02	2.9	1.2
5-FdUrd	0.0003	0.0008	2.6	0.003

**Table 16:** The antitumor activities of the pyrimidine and purine derivatives of ASA.

Comp.	IC_50_ (μM)								
	L1210/0	Molt4/C8	CEM/0	HeLa	MCF-7	MiaPaCa-2	Hep-2	SW 620	WI 38
**(Z)-41b**, R^5^ = F	5.2	118	77.4	50	59.3	58.1	>100	>100	42.6
**(Z)-41c**, R^5^ = CF_3_	>200	>200	>200	5.6	8.8	12.8	5.6	8.8	11.6
**44a**, R^6^ = Cl	21.8	19.8	22.9	6.8	14.3	6.5	14.8	20	16.1
5-FU	0.69	20	9.23	16	4.5	6.5	51	8.7	10
ASA	>200	>200	>200	>200	>200	>200	>200	>100	>200
DiBnASA	199.4	143.2	151.7	>100	>200	>100	>200	>100	>100

**Table 17:** The cytostatic activities of C-5 aryl-, alkenyl-, and alkynyl-substituted uracil derivatives of ASA.

Comp.	IC_50_ (μM)								
	L1210	Molt4/C8	CEM/0	HeLa	MCF-7	MiaPaCa-2	Hep-2	SW 620	WI 38
**45a**, R^5^ = propynyl	0.47	0.78	0.77	0.2	0.2	0.5	0.7	0.3	0.2
**45b**, R^5^ = furyl	6.8	6.6	7.0	2	2	1.6	2.2	2.4	1.8
**45c**, R^5^ = vinyl	7.2	7.6	7.2	14.5	3.8	22	14.2	15.6	14.3
**45d**, R^5^ = ethynyl	6.8	7.2	4.6	3.2	2.9	8.7	4.6	5	1.5
**45e**, R^5^ = isopentenyl	8.2	8.3	7.7	2	4	5	2.6	4	2
**46a**, R^5^ = ethynyl-Ph	8.1	8.2	7.6	2	1	3	3	4	1

**Table 18:** The antiviral activity of the C-5-alkynyl uracil ASA derivative.

Comp.	EC_50_ (μM)			CC_50_(μM) Cytotoxicity
	Sindbis Virus	Coxsackie Virus B4	Vesicular Stomatitis Virus	
**45a**, R^5^ = propynyl	1.6	1.6	1.6	8
Ribavirin	100	>500	100	>500

**Table 19:** The cytostatic activities of C-5 substituted pyrimidine and 6-alkylfuro[2–3-*d*]pyrimidine-4,5-didehydro-l-ASA derivatives.

Comp.	IC_50_ (μM)							
	L1210/0	Molt4/C8	CEM/0	HeLa	MiaPaCa-2	SW 620	MCF-7	H-460
**47a**, R = hexyl	6.8	3.0	2.0	12	3	4	4	2.4
**47b**, R = 4-Br-Ph	10	15	7.6	8	13	7	21	11
**47c**, R = 4-CH_3_-Ph	8.7	37	9.6	14	21	18	19	23
**47d**, R = 4-butyl-Ph	8.2	9.3	8.3	4	16	7	17	12
**47e**, R = 4-pentyl-Ph	8.0	6.9	6.6	3	10	6	9	NT ^a^
**48a**, R = butyl	4.5	9.0	7.7	17	15	16	16	16
**48b**, R = 4-Br-Ph	9.5	9.6	8.3	20	14	20	13	16

^a^ NT = not tested.

**Table 20:** The antiviral activities of C-5 substituted pyrimidine-4,5-didehydro-l-ASA derivatives.

Comp.	EC_50_ (μM)	Cytotoxicity CC_50_ (μM)
	CMV (Davis)	
**47a**, R = hexyl	1.8	10
**47c**, R = 4-CH_3_-Ph	3.8	>20.4
Ganciclovir	2.6	262
Cidofovir	0.67	133

**Table 21:** The cytostatic activities of the cytosine (**49a**) and 5-azacytosine (**49b**) ASA derivatives.

Comp.	IC_50_ (μM)					
	HeLa	MCF-7	HepG2	SW 620	MiaPaCa-2	WI 38
**49a**	7.20	2.03	1.98	>100	24.24	8.49
**49b**	5.91	3.49	2.72	5.62	0.92	4.67

**Table 22:** The cytostatic activities of 9-deazapurine ASA derivatives.

Comp.	IC_50_ (μM)						
	L1210/0	CEM/0	HeLa	MiaPaCa-2	Hep-G2	SW620	3T3
**57**	4.7	4.1	12	33.9	>100	47.1	>100
**(Z, Z)-59**	4.5	19	5.6	26.2	>100	47.2	>100
5-FU	NT ^a^	NT ^a^	66.5	11.67	55.2	0.79	28.3

^a^ NT = not tested.

**Table 23:** The antiviral activity of the 3-deazapurine derivative **54a**.

Comp.	EC_50_ (μM) HCMV		CC_50_ (μM)
	AD-169	Davis Line	HeLa
**54a**, R^1^ = R^3^ = F, R^2^ = Cl, R^4^ = CH_2_Ph	8.94	8.94	>100
Ganciclovir	6.12	4.72	328.5

**Table 24:** The anticancer and anti-varicella-zoster virus (VZV) activity of purine and butenolide conjugates.

Comp.	IC_50_ (μM)					CC_50_ (μM)	IC_50_ (μM)
	L1210/0	P388	MCF-7	Molt4/C8	CEM/0	Hef522	TK-VZV (YS/R)
**60a**	4.52	8.12	16.87	5.97	4.71	30.47	1.62
**60b**	5.81	6.49	15.43	7.35	3.98	27.86	1.03
**60c**	6.03	7.42	14.21	6.16	5.04	32.21	0.89
**61**	93.35	2.67	>120	78.46	84.51	93.80	6.07
**64c**	1.03	0.28	0.76	1.74	0.98	17.40	-
ara-C	0.17	0.14	1.03	0.65	0.78	2.14 × 10^−2^	-
Acyclovir	4.52	-	-	-	-	-	26.43

**Table 25:** The inhibitory activity of purine and butenolide conjugates toward *Escherichia coli* ribonucleotide diphosphate reductase (RDPR) enzyme.

Comp.	*E. coli* RDPR inhib. (%) ^a^	
	100 μM	1000 μM
**60a**	59.47	9.94
**60b**	58.96	11.07
**60c**	55.34	10.20
**63a**	>99	>99
**64c**	<1	0

^a^ Enzyme activity remaining (%).

**Table 26:** The antiviral activities of lipophilic prodrugs **66**–**68** and their parent molecules.

Comp.	LD_50_ (mg/kg)	IC_50_ (μg/mL)			
	HSV-1	HIV-1	HIV-2	MSV	MT4
**66**	710	1.4	1.0	0.93	265
**67**	-	6.0	7.1	0.02	14
**68**	675	4.9	4.2	13	>300
PMEA	-	4.1	3.8	2.0	274
PMEG	-	16	18	0.19	16

**Table 27:** The antitumor and anti- hepatitis C virus (HCV) activities of the 4,5-disubstituted-imidazole ASA derivatives.

Comp.	IC_50_ (μM)		EC_50_ (μg/mL)	CC_50_ (μg/mL)
	CEM/0	WI 38	Hepatitis C	MT4
**71a**	10	>100	13.3	70.5
**71b**	7.3	73	>100	>100
Doxorubicin	0.39	0.04	-	-

**Table 28:** The anti-influenza activity of the pentacyclic triterpene ASA conjugate **72b**.

Comp.	EC_50_ (μM)	CC_50_ (μM)
	A/WSN/33	
**72b**	8.7	>200
Oseltamivir	12.5	>200

**Table 29:** The antproliferative effect of butenolides substituted with 1,3,4-thiadiazoles (**74a**–**l**) or 1,2,4-triazole Schiff bases (**75a**–**l**) on HeLa cell line.

Comp.	IC_50_ (μM)	Comp.	IC_50_ (μM)
	HeLa		HeLa
**74a**, R = Ph	3.0	**75a**, R = Ph, R^2^ = 4-Cl-Ph	19.7
**74b**, R = 4-Cl-Ph	2.2	**75b**, R = 4-OCH_3_-Ph, R^2^ = 4-Cl-Ph	4.4
**74c**, R = 2-OH-Ph	2.9	**75c**, R = Ph, R^2^ = 4-NO_2_-Ph	11.6
**74d**, R = 4-OH-Ph	2.6	**75d**, R = 4-OCH_3_-Ph, R^2^ = 4-NO_2_-Ph	11.2
**74e**, R = 4-NO_2_-Ph	0.9	**75e**, R = Ph, R^2^ = 2-furyl	6.8
**74f**, R = 4-OCH_3_-Ph	3.7	**75f**, R = 4-OCH_3_-Ph, R^2^ = 2-furyl	5.1
**74g**, R = 2-furyl	1.7	**75g**, R = CH_3_, R^2^ = 4-Cl-Ph	8.2
**74h**, R = pyridine-4-yl	1.3	**75h**, R = 4-OH-Ph, R^2^ = 4-OCH_3_-Ph	7.1
**74i**, R = pyridine-3-yl	5.6	**75i**, R = Ph, R^2^ = 4-OH-Ph	3.7
**74j**, R = 2-furyl	1.3	**75j**, R = Ph, R^2^ = 4-Cl-Ph	4.5
**74k**, R = pyridine-4-yl	3.0	**75k**, R = 4-OH-Ph, R^2^ = 4-OCH_3_-Ph	6.2
**74l**, R = pyridine-3-yl	3.4	**75l**, R = CH_3_, R^2^ = 2-OH-Ph	1.8
		Cisplatin	2.6

**Table 30:** The antitumor activities of butenolides **76** with a dithiocarbamate side chain.

Comp.		IC_50_ (μM)	Comp.		IC_50_ (μM)
		HeLa			HeLa
**76a**, R =	(CH_2_CH_3_)_2_N-	1.39	**76f**, R =	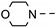	2.43
**76b**, R =	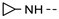	24.14	**76g**, R =	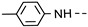	5.44
**76c**, R =	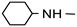	150.03	**76h**, R =	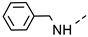	15.08
**76d**, R =	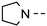	2.63	5-FU		41.46
**76e**, R =	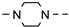	0.77			

**Table 31:** The antitumor activities of dysidiolide derivatives.

Comp.	IC_50_ (μM)
	HeLa, A-549, HT-29, HL-60
**79b**, (3′*R*)	3.3–11.1
**80a**, (3′*S*)	3.2–4.8
**80b**, (3′*R*)	3.3–5.4
**81a**, (3′*S*)	2.6–3.5
**81b**, (3′*R*)	2.9–3.5
**82a**, (3′*S*)	3.0–3.8
**82b**, (3′*R*)	2.3–3.8
**83**	0.3–3.1

**Table 32:** The antitumor activities of the digoxin derivative.

Comp.	IC_50_ (μM)		
	HeLa	RKO	WI-26 VA4
(Z)-**85a**, R = 4-N(CH_3_)_2_	0.26	0.48	0.65
Digoxin	2.2	0.42	NT ^a^

^a^ NT = not tested.

**Table 33:** The anti-influenza A (H1N1) activity of aspulvinone derivatives.

Comp.	EC_50_ (μg/mL)	CC_50_ (μg/mL)
	Influenza A (H1N1)	A549; MDCK
**(*E*)-86**	32.3	>250
**(*Z*)-86**	56.9	>250
**87**	29.1	>250
Ribavirin	24.6	
Zanamivir	28.4	

## Supplementary Materials

The following are available online at https://www.mdpi.com/2076-3921/8/8/247/s1, Figure S1: ASA derivatives with OH groups modified by alkyl groups; Figure S2: The 6-Palmi-ASA-2P as a ROS scavenger; Figure S3: The 5-O-modified ASA derivative with a radical scavenging activity; Figure S4: The C-6 substituted ASA derivatives; Figure S5: The structure of 3-O-ethylascorbic acid; Figure S6: γ-(Triazolylethylidene)butenolides; Figure S7: The butenolide diterpene isolated from the *Metaporana sericosepala*.
