# Supplementary material for: Therapeutic Perspective of Vitamin C and Its Derivatives
Source: Antioxidants (Basel). 2019 Jul 26;8(8):247. doi: 10.3390/antiox8080247 (PMC6721080; doi:10.3390/antiox8080247)
Supplement: Supplementary file 1 [file antioxidants-08-00247-s001.pdf]

Supplementary Information for:

Review

Therapeutic Perspective of Vitamin C and Its Derivatives

Andrijana Meščić Macan[1,*], Tatjana Gazivoda Kraljević[1] and Silvana Raić-Malić[1]

[1] 1 Department of Organic Chemistry, University of Zagreb, Faculty of Chemical Engineering and Technology, Marulićev trg 20, 10 000 Zagreb, Croatia

* Correspondence: amescic@fkit.hr; Tel.: +385-1-4597203)

Contents:

Figures S1–S7 **S1–S2**

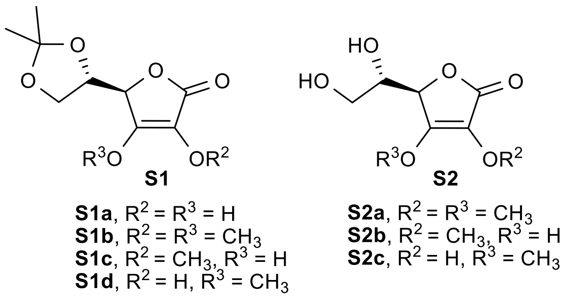

Figure S1. ASA derivatives with OH groups modified by alkyl groups.

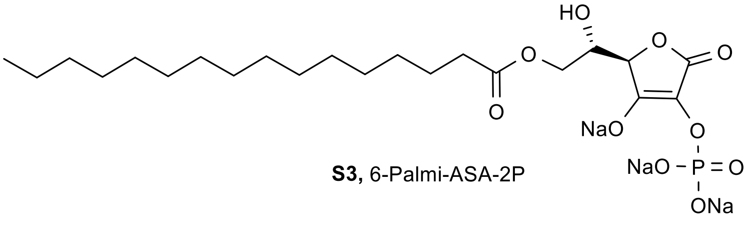

Figure S2. The 6-Palmi-ASA-2P as a ROS scavenger.

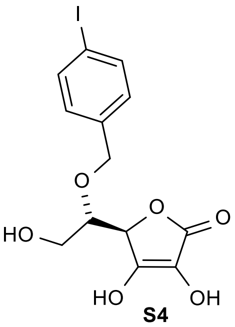

S4

Figure S3. The 5-*O*-modified ASA derivative with a radical scavenging activity.

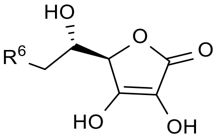

S5a, R^6 = Cl
S5b, R^6 = Br
S5c, R^6 = NH_2
S5d, R^6 = $N(CH_3)_2$

Figure S4 The C-6 substituted ASA derivatives.

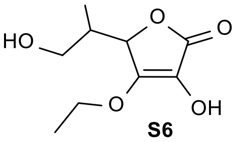

S6

Figure S5. The structure of 3-*O*-ethylascorbic acid.

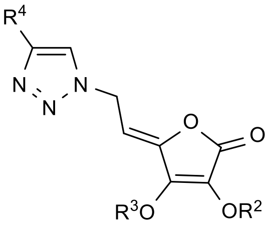

S7

$R^2 = R^3 = CH_2Ph, CH_3$
R^4 = alkyl, Ph, OH-alkyl, CH_2-Ph, 4-alkyl-Ph, pyridine-2-yl, cyclohexenyl, cyclopropyl

Figure S6. γ-(Triazolylethylidene)butenolides.

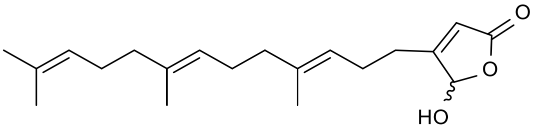

S8, IC_{50} (A2780) = 8 μM

Figure S7. The butenolide diterpene isolated from the *Metaporana sericosepala*.
